# Prophylactic Radical Fimbriectomy with Delayed Oophorectomy in Women with a High Risk of Developing an Ovarian Carcinoma: Results of a Prospective National Pilot Study

**DOI:** 10.3390/cancers15041141

**Published:** 2023-02-10

**Authors:** Eric Leblanc, Fabrice Narducci, Gwenaël Ferron, Audrey Mailliez, Jean-Yves Charvolin, El Hajj Houssein, Frédéric Guyon, Virginie Fourchotte, Eric Lambaudie, Agathe Crouzet, Yves Fouche, Sébastien Gouy, Pierre Collinet, Frédéric Caquant, Christophe Pomel, François Golfier, Véronique Vaini-Cowen, Isabelle Fournier, Michel Salzet, Emmanuelle Tresch, Alicia Probst, Anne-Sophie Lemaire, Marie-Cécile Le Deley, Delphine Hudry

**Affiliations:** 1 Department of Gynecologic Oncology, Centre Oscar Lambret, 3 rue Frédéric Combemale, 59020 Lille, France; f-narducci@o-lambret.fr (F.N.); houssein.elhajj@curie.fr (H.E.H.); d-hudry@o-lambret.fr (D.H.); 2 INSERM U1192, Université de Lille, 59000 Lille, France; isabelle.fournier@univ-lille.fr (I.F.); michel.salzet@univ-lille.fr (M.S.); 3 Centre Claudius Regaud, 31300 Toulouse, France; ferron.gwenael@iuct-oncopole.fr; 4 Medical Oncology Department, Centre Oscar Lambret, 59020 Lille, France; a-mailliez@o-lambret.fr; 5 Clinique du Bois, 59000 Lille, France; charvolin@charvolin.com; 6 Institut Jean-Alban Bergonié, 33076 Bordeaux, France; f.guyon@bordeaux.unicancer.fr; 7 Institut Curie, 75248 Paris, France; virginie.fourchotte@curie.net; 8 Institut Paoli-Calmettes, 13009 Marseille, France; lambaudiee@ipc.unicancer.fr; 9 Centre Henri Becquerel, 76038 Rouen, France; agathe.crouzet@chb.unicancer.fr; 10 Centre Antoine Lacassagne, 06100 Nice, France; yves.fouche@nice.unicancer.fr; 11 Institut Gustave Roussy, 94805 Villejuif, France; sebastien.gouy@gustaveroussy.fr; 12 Hôpital Jeanne de Flandre, CHRU, 59000 Lille, France; pierre.collinet@chru-lille.fr; 13 Hôpital Privé de Villeneuve d’Ascq, 59650 Villeneuve-d’Ascq, France; frederic.caquant@mac.com; 14 Centre Jean Perrin, 63011 Clermont-Ferrand, France; christophe.pomel@cjp.fr; 15 Hospices Civils de Lyon, 69002 Lyon, France; francois.golfier@chu-lyon.fr; 16 Polyclinique du Parc Rambot, 13100 Aix en Provence, France; dr.vve@wanadoo.fr; 17 Biostatistics Unit, DRCI, Centre Oscar Lambret, 59020 Lille, France; scientifique@o-lambret.fr; 18 Sponsor Unit, DRCI, Centre Oscar Lambret, 59020 Lille, France; a-probst@o-lambret.fr (A.P.); m-ledeley@o-lambret.fr (M.-C.L.D.); 19 Pathology Department, Centre Oscar Lambret, 59020 Lille, France; a-lemaire@o-lambret.fr; 20 Centre de Recherche en Epidémiologie et Santé des Populations, INSERM, Paris-Sud, Paris-Saclay University, 94800 Villejuif, France

**Keywords:** early menopause, delayed oophorectomy, high risk of breast and ovarian cancer, ovarian cancer prevention, radical fimbriectomy, risk-reducing surgery

## Abstract

**Simple Summary:**

Risk-reducing salpingo-oophorectomy is the gold standard for the prophylaxis of ovarian cancer in high-risk women. However, 20–30% of women delay or refuse early oophorectomy due to significant adverse effects related to induced early menopause. We performed a pilot study to evaluate a two-step ovarian cancer risk-reducing approach with radical fimbriectomy followed by a delayed oophorectomy. A total of 121 patients underwent radical fimbriectomy. This approach appears to be safe, well tolerated, and avoids surgery-induced early menopause, while no high-grade serous adnexal carcinomas have been reported to date in this cohort with a median follow-up of 7.3 years.

**Abstract:**

Risk-reducing salpingo-oophorectomy is the gold standard for the prophylaxis of ovarian cancer in high-risk women. Due to significant adverse effects, 20–30% of women delay or refuse early oophorectomy. This prospective pilot study (NCT01608074) aimed to assess the efficacy of radical fimbriectomy followed by a delayed oophorectomy in preventing ovarian and pelvic invasive cancer (the primary endpoint) and to evaluate the safety of both procedures. The key eligibility criteria were pre-menopausal women ≥35 years with a high risk of ovarian cancer who refused a risk-reducing salpingo-oophorectomy. All the surgical specimens were subjected to the SEE-FIM protocol. From January 2012 to October 2014, 121 patients underwent RF, with 51 in an ambulatory setting. Occult neoplasia was found in two cases, with one tubal high-grade serous ovarian carcinoma. Two patients experienced grade 1 intraoperative complications. No early or delayed grade ≥3 post-operative complications occurred. After 7.3 years of median follow-up, no cases of pelvic invasive cancer have been noted. Three of the fifty-two patients developed de novo breast cancer. One BRCA1-mutated woman delivered twins safely. Twenty-five patients underwent menopause, including fifteen who had received chemotherapy for breast cancer, and twenty-three underwent menopause before the delayed oophorectomy, while two did not undergo a delayed oophorectomy at all. Overall, 46 women underwent a delayed oophorectomy. No abnormalities were found in any delayed oophorectomy specimens. Radical fimbriectomy followed by delayed oophorectomy appears to be a safe and well-tolerated risk-reducing approach, which avoids early menopause for patients with a high risk of breast and ovarian cancer.

## 1. Introduction

Ovarian carcinoma (OC) is the 8th most common cause of cancer in women worldwide, with 313,959 new cases in 2020, and is, by far, the most lethal gynecologic cancer [1].

Most OCs are considered sporadic, with a lifetime risk of 1.3% (1/78 women). A family history of breast and/or ovarian cancer mostly related to a genetic mutation is responsible for almost 18% of OC cases [2]. Of these genetic mutations, BRCA1 and BRCA2 gene mutations (implicated in the process of the homolog recombination repair of DNA double-strand breaks) are the most frequently associated with an increased risk of adnexal carcinoma [3]. 

The lifetime risk of developing an OC, especially a high-grade serous subtype (HGSC), varies from 16% to 68% in the case of the BRCA1 mutation and from 11% to 30% in the case of the BRCA2 mutation [4]. This risk increases exponentially with time, from lower than 3% for patients under 40 years old, rising to 10% by the age of 50 to reach 49% and 21% for the BRCA1 and BRCA2 mutations, respectively, by the age of 80 [5].

Large, randomized trials (PLCO and UK-TOCS) confirmed that current screening methods are disappointing in their ability to detect early-stage ovarian cancers and thus decrease mortality [6,7], even when focusing on this high-risk subgroup [8]. Thus, the role of prevention is essential to reduce the incidence of this dreadful disease. Medical methods, such as combined contraceptive pills, aspirin, and metformin, have shown variable levels of protection [9]. Yet, they are not always indicated or well-tolerated, and, in the long run, they are less efficient than risk-reducing surgery [10]. 

Since the early 2000s, risk-reducing salpingo-oophorectomy (RRSO) has become the standard procedure for OC prophylaxis, particularly after reporting high rates of tubal epithelial abnormalities in the surgical specimens of BRCA-mutated patients undergoing RRSO [11]. This was rapidly confirmed after adopting the sectioning and extensively examining the fimbriated end (SEE-FIM) protocol for all RRSOs [12]. SEE-FIM, a more precise examining protocol [13], highlighted the fimbria as the predilection site for tubal epithelial abnormalities [14] and proposed the hypothesis that most HGSCs might arise from the mesothelial–Müllerian junction [15,16] and that other OC subtypes arise from the tubo–ovarian junction [17]. Experimental studies confirmed the tubal origin of HGSCs [18] and proposed several models of tumor development [19,20,21]. 

RRSO performed at a younger age is frequently associated with significant adverse effects [22] that can only be partially balanced with hormone replacement therapy [23,24]. In addition, this option is contraindicated in patients with breast cancer history. For these reasons, up to 30% of BC women delay or refuse RRSOs [25].

These findings led to the idea of a two-step prophylactic procedure consisting of a bilateral risk-reducing radical fimbriectomy followed by a delayed oophorectomy (RF/DO). Rising interest in this option requires further evaluation [26].

In 2012, we began a prospective national pilot study evaluating RF/DO in France. It aimed to assess the efficacy of RF/DO at preventing ovarian and pelvic HGSCs and assess the safety of both procedures. 

## 2. Methods

This study’s protocol, NCT-01608074, was approved by the institutional review board and by an independent ethics committee.

### 2.1. Eligibility Criteria

The inclusion criteria were as follows: non-pregnant, pre-menopausal women aged 35 years old or more, who have completed family planning, presented a documented HBOC (BRCA1 or BRCA2 mutations or history of breast/ovarian cancer in first-degree relatives), and have refused RRSOs. RRSO was always proposed first, as recommended by our national guidelines. 

### 2.2. Study Procedures and Follow-Up (Supplementary Materials)

Radical fimbriectomy (RF) consists of the complete resection of both fallopian tubes from their uterine insertion to their fimbrial extremity, along with the small ovarian portion adherent to the fimbria in order not to miss the pathological examination of the tubo–ovarian junction [27]. DO was recommended systematically at the age of 50 or at menopause, if it occurred earlier, in case of other medical indications, or at the patient’s wish. RF and DO were generally performed by minimal-invasive approaches (regular, single/multiport laparoscopy, or even by a robotic approach). 

Before their enrollment, all patients underwent a complete physical and biological workup. The baseline biological workup included the tumor marker (CA125) and hormone levels. The RF/DO protocol was thoroughly explained to the patients, who all signed the informed consent before their inclusion. 

Before enrollment, all patients were screened for their menopausal status, which was defined by the absence of menses for at least 12 months, associated with an elevated FSH and/or low estrogen levels. 

The standard pathologic examination of all surgical specimens was followed for FR by the SEE-FIM protocol. HES staining and p53-Ki67 immunochemistry were performed to characterize the different pathological aspects (p53 signature, serous tubal intraepithelial lesion (STIL), serous tubal intraepithelial carcinoma (STIC), or a true invasive carcinoma) [28]. Furthermore, in the case of doubt, a centralized review of specimens was performed by the French group of pathologists.

A yearly follow-up was performed until the oophorectomy and included a clinical evaluation and tumor marker assessment.

### 2.3. Study Endpoints

To estimate the cumulative incidence of ovarian and pelvic invasive cancer after RF, the primary endpoint was the time interval between the RF and the occurrence of ovarian or pelvic invasive cancer. Death without the occurrence of ovarian or pelvic invasive cancer was considered a competing event. Data were censored at the last follow-up visit in patients alive without ovarian or pelvic invasive cancer.

Secondary endpoints were the evaluation of the RF-related morbidity; the incidence and the morbidity of DO; the prevalence of incidental in situ or invasive lesions found on surgical specimens; the occurrence of menopause at the last follow-up and before DO, if any; and the evaluation of breast cancer incidence after RF.

All intraoperative and post-operative complications were described. The Clavien–Dindo classification [29] was used to grade the 30-day post-operative morbidity after RF and DO. The NCI-CTCAE v4 classification was used to grade delayed medical and surgical adverse events (AEs) classified as related to the procedures.

### 2.4. Statistical Considerations

This pioneering study was designed with preplanned safety-stopping rules to detect an increased incidence of ovarian carcinoma. We assumed a cumulative incidence of ovarian carcinoma of 10% at 10 years in the absence of prophylactic surgery, which was grossly translated into a 3% cumulative incidence at 3 years, regardless of the gene mutation, knowing that the risk varies with the age and the underlying genetic mutation [30,31]. Per protocol, a dynamic method for the interim analyses of rare events was used to discontinue the study if an excess of pelvic tumors was observed, considering the comparison of the estimated 3-year cumulative incidence to the maximum acceptable value *p* = 3%, tested at a one-sided alpha level of 10% with a maximum sample size set at 120 patients [32].

The cumulative incidence of ovarian/pelvic invasive cancer was estimated using the Kalbfleish and Prentice method [33], considering death without the considered event as a competing event. A similar approach was used to estimate the cumulative incidence of de novo breast cancer and secondary oophorectomy. Estimates are displayed with their 95% confidence intervals (95% CI). Follow-up was estimated using the inverse Kaplan–Meier method.

All patients who underwent RF are included in the safety analyses. The incidences of secondary pelvic cancer, menopause, and DO were estimated, excluding patients with invasive ovarian/pelvic cancer or a STIC lesion on the RF surgical specimen. The incidence of de novo breast cancer was estimated in patients without a prior personal history of breast cancer.

An interim analysis was performed in January 2014 and reviewed by an independent data monitoring committee, which recommended the study’s continuation.

## 3. Results

**Figure 1:**
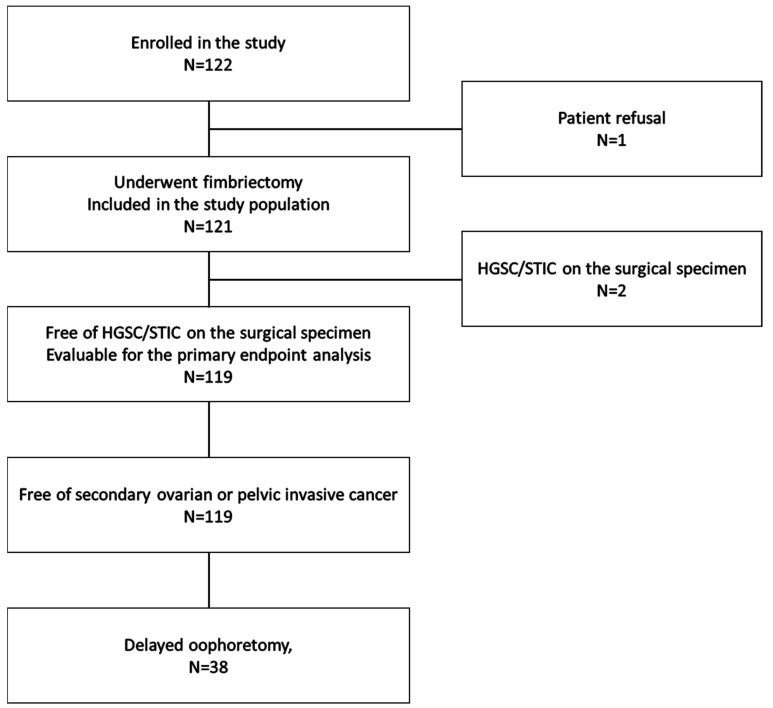
Participant flow.

From January 2012 to October 2014, 122 patients from 14 hospitals were enrolled in the trial. Overall, 121 patients underwent RF and are included in the current analysis. One patient opted out after inclusion and decided to undergo RRSO. The details of the participating patients are shown in the flowchart in Figure 1.

Baseline Characteristics (Table 1)

Overall, 76 (62.8%) women presented a BRCA1 mutation, 31 (25.6%) displayed a BRCA2 mutation, and 14 women showed an HBOC with no identified genetic mutation. The median age at inclusion was 39 years (range, 28–48) for women with a BRCA1 mutation, 40 years (35–44) for women with a BRCA2 mutation, and 41 years (37–46) for the rest of the cohort. 

None of the patients were menopausal at the inclusion time. However, in 11 women, their menses were irregular, which was related to their breast cancer treatment (chemotherapy or LHRH-agonists).

Thirty patients had had a prior bilateral prophylactic mastectomy.

Surgical Data (Table 2)

### 3.1. Radical Fimbriectomy

All except one RF were performed by laparoscopy. The median operative time was 45 min (18–262 min), the median estimated blood loss was 50 mL (4–250 mL), and the median length of stay was one night (0–31 nights). In 51 cases (42.1%), the surgery was performed in an ambulatory setting.

In 93.2% of the cases, the ovarian segment was transected sharply, using cold scissors to avoid tissue damage. Hemostasis was performed using intermittent bipolar current applications. All the ovaries had a normal non-ischemic appearance at the end of the procedure.

### 3.2. Delayed Oophorectomy

**Figure 2:**
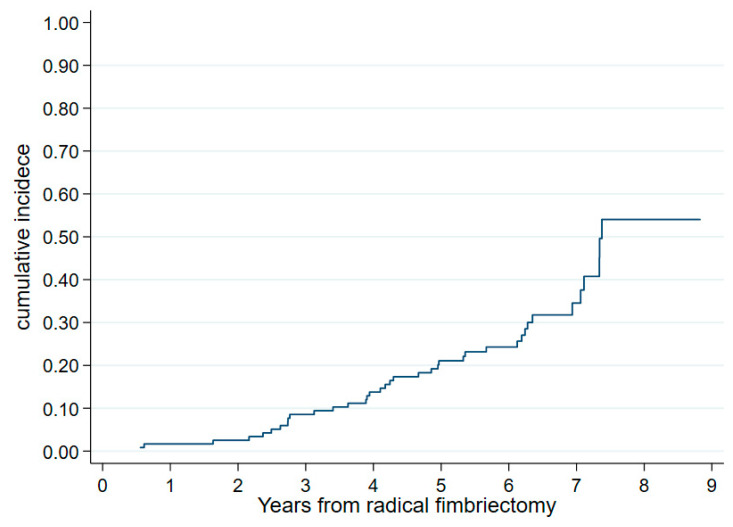
Cumulative incidence of oophorectomy from radical fimbriectomy (N = 119).

Overall, 46 of the 119 evaluable patients underwent delayed bilateral oophorectomy (DO) at a median age of 46 years (39–52). The reasons for DO were (a) menopause in twenty-three patients, reaching the age of 50 in four patients, (b) other medical reasons in eleven patients (an increase in the CA125 in one patient, dysmenorrhea in three patients, metrorrhagia in four patients, ovarian cysts in two patients, and the presence of a P53 lesion on the surgical specimen in one patient), and (c) following the patient’s wishes in eight patients. The 6-year cumulative incidence of DO was 22.6% (a 95% CI, 16.0–31.4%) (Figure 2). The procedure was performed by laparoscopy in forty-one and laparotomy in three patients (we are missing data for two patients). Nine patients had an associated total hysterectomy.

### 3.3. Morbidity 

#### 3.3.1. Early Morbidity

During the RF procedure, two patients experienced grade 1 complications (bleeding that was managed laparoscopically and did not require a transfusion).

After the RF, 24 patients (19.8%) experienced at least one AE during the 30 post-operative days. A Clavien–Dindo grade 1 complication was seen in twenty-two patients, and a grade 2 complication occurred in two patients. No serious adverse events or hospital re-admissions were reported. 

One of the patients who underwent a laparotomy for a hysterectomy with a concomitant DO presented immediate post-operative vaginal bleeding from a uterine pedicle, requiring re-operation and a blood transfusion. No post-operative grade 2–3 complications were reported after the DO.

#### 3.3.2. Late Morbidity

No late surgery-related AE (≥30 days) was found after the RF or DO, except for one case of grade 2 dysmenorrhea. 

During a follow-up, one patient presented an ovarian torsion caused by a cyst (an independent, not surgery-related event). This required a unilateral oophorectomy completed by a contralateral delayed oophorectomy after the age of 50.

#### 3.3.3. Menopause

At the last follow-up and before the DO, if any, twenty-five of the one hundred and nineteen (21.0%) patients were clinically and biologically menopausal, with sixteen of seventy-four patients displaying a BRCA1 mutation, eight of thirty-one patients displaying a BRCA2 mutation, and one of fourteen patients with no identified genetic mutation. Twelve of these patients received chemotherapy for breast cancer before the RF and three after the RF. 

The median age of menopause was 44.8 years (39.5–50.8), with two cases of menopause before 40 years; one patient had had previous breast cancer-related chemotherapy, whereas the other patient had no other identifiable risk factors explaining the menopause, except for the RF.

Pathological Results (Table 3)

Two patients (1.7%, a 95% CI, 0.2–5.8%) presented incidental pathologic findings. One patient presented a 3 mm tubal HGSC with a contralateral tubal STIC lesion and positive cytology. She underwent surgical staging and was upstaged to stage II ovarian cancer due to parametrial involvement. This was followed by six cycles of adjuvant carboplatin-paclitaxel chemotherapy. One hundred and one months after her RF, this patient was being treated with PARP inhibitors maintenance therapy and did not present any evidence of disease recurrence.

The second patient presented a unilateral tubal STIC lesion with negative cytology. She underwent peritoneal staging and bilateral oophorectomy with normal pathologic analysis. After a follow-up of 77 months, this patient did not present signs of recurrence.

None of the other patients were re-operated on for tubal abnormalities, and all of the ovarian fragments were disease-free.

Overall, a tubal lesion (HGSC/STIC/STIL/p53 lesion) or abnormality (≥1 site with Ki67 > 10%) was observed in 25 patients (20.7%). It is important to note that none of the tubal abnormalities (invasive or not) were observed at the mesothelial–Müllerian or tubo–ovarian junctions. We also did not observe any significant relationship between the BRCA status and the occurrence of tubal lesions (fifteen of seventy-six, seven of thirty-one, and three of nine in the BRCA-mutated, BRCA2-mutated, and non-mutated, respectively (Fisher’s exact test, *p* = 0.58).

No malignancies were found on the surgical specimens of the 46 patients who underwent a DO.

### 3.4. Follow-Up

#### 3.4.1. Ovarian and/or Pelvic Carcinoma

With a median follow-up of 7.3 years from the date of the RF (interquartile range: 6.0–8.3 years, max: 9.9 years), no ovarian or pelvic HGSC was reported among the 119 evaluable patients. The only pelvic oncologic lesion was a grade 3 cervical intraepithelial neoplasia (CIN3), incidentally discovered 5.6 years after the RF, and it was treated with a total hysterectomy without oophorectomy. Thus, the cumulative incidence of ovarian/pelvic invasive carcinoma is 0%. 

#### 3.4.2. Breast Cancer 

**Figure 3:**
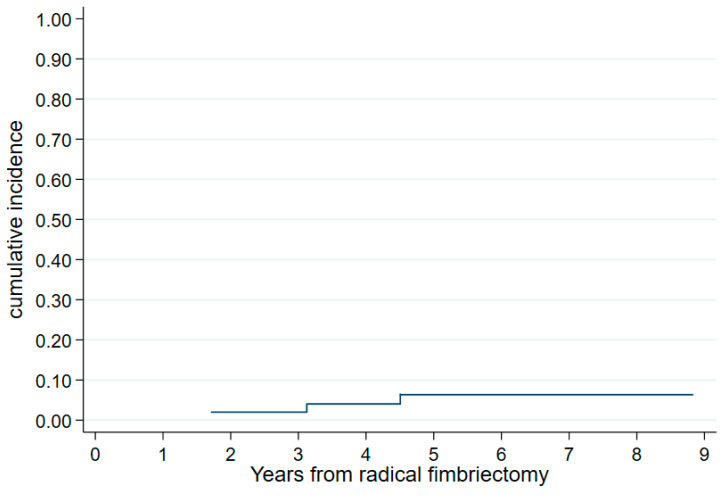
Cumulative incidence of breast cancer from radical fimbriectomy in patients with no personal history of breast cancer (N = 52).

At the last follow-up, three of the fifty-two patients with no prior breast cancer and no HGSC/STIC lesion had developed breast cancer: triple-negative tumors, all in the BRCA1 mutation carriers, and all within the six years after the RF leading to a 6-year cumulated incidence of newly diagnosed breast cancer of 6.4% (95% CI, 2.1–18.9%) (Figure 3). None of the patients had had a secondary oophorectomy before this event. In addition, 20 of the 69 patients who had a prior history of breast cancer had a relapse, leading to death in two patients (BRCA1- and BRCA2-mutated). 

#### 3.4.3. Other

A 36-year-old patient, BRCA1-mutated, gravida1 para1, had a laparoscopic RF with no suspicious lesions or pathology. One year later, she underwent assisted reproductive technology to become pregnant and delivered healthy twins at 35 weeks of pregnancy. 

## 4. Discussion

RF/DO is a new two-step surgical prophylactic procedure to prevent ovarian carcinoma. It is based on the pathological findings that conclude the tubal origin of the OC, particularly in the case of BRCA 1 or 2 mutations and HBOC. However, contrary to the standard RRSO procedure, this new alternative aims to preserve ovarian function until natural menopause, thus avoiding early menopause-related effects. Radical fimbriectomy was preferred to simple salpingectomy to analyze the mesothelial–Müllerian (tubo– and peritoneo–ovarian) junctions that are supposedly at the origin of OC precursors [16]. However, due to the absence of abnormalities at these levels, a simple total bilateral salpingectomy might replace radical fimbriectomy.

We believed that a prospective controlled trial was necessary to assess the effectiveness of this strategy; therefore, we offered it to all patients with HBOC who refused the RRSO and met the inclusion criteria. 

As it was the first prospective study on a new two-step prophylactic procedure in high-risk women, it was designed on a relatively small sample with stopping rules to closely monitor and warrant oncological safety. However, it already shows a prophylactic effect on the development of pelvic HGSC, as no such case has been observed in this cohort so far, even after a median follow-up of more than seven years. Patient surveillance is still ongoing, and a 10-year assessment is already planned. 

Although these results are promising, a more extensive international observational study comparing the standard RRSO to radical fimbriectomy or simple salpingectomy and delayed oophorectomy is necessary to confirm the non-inferiority of these less aggressive strategies in terms of adnexal cancer prophylaxis with at least a 10-year follow-up. This choice of method should be the decision of the individual women, as proposed in the new TUBA-WISPII study (NCT04294927). Offering these women at high risk such a choice would be an excellent means of decreasing the significant rate of young women reluctant to undergo such an operation [25] and, consequently, exposing themselves to this fearsome disease. 

The peri-operative morbidity of both procedures (RF and DO) was minimal and comparable to the RRSO-related morbidity [34,35].

Out of the 25 of 119 patients who underwent menopause after the RF, only two cases occurred before the age of 40. Despite the objective impact of the previous breast cancer-related chemotherapy on ovarian reserves [36,37], we cannot rule out a possible impact of the infundibulopelvic (IP) ligament hemostasis during the RF. IP coagulation with resultant relative ischemia might accentuate the reduction of the ovarian reserve, already impacted by the BRCA mutation [38], and accelerate the onset of menopause. This effect was not observed in the hysterectomy-salpingectomy for benign pathologies [39]. The TUBA non-randomized prospective study compared HBOC patients undergoing RR salpingectomy (RRS) and RRSO. It clearly demonstrated that the quality of life after RRS was superior to RRSO, even when taking into account hormonal replacement therapy [40]. Another project (PROTECTOR), with sexual function as the primary objective, has been recently initiated [41].

Our rate of incidental HGSC or STIC lesions was 1.7% (two patients, with a total of three lesions). This could be deemed low but is in the range of those observed in the literature [42] and, either way, exceeds the rate observed in a low-risk population by at least ten times [43]. The young median age of our patients is likely the reason for observing fewer pathological abnormalities when compared to that of the literature [35]. In addition, in the literature data, only 11–61% of HGSC ovarian carcinomas were associated with STICs [44]. Thus, the possibility of an intermediate cellular precursor cannot be ruled out, given its longer time intervals when developing into a carcinoma [45], suggesting a possible advantage of performing such a procedure earlier than is currently recommended, namely as soon as the person in question no longer wishes to be pregnant. 

Interestingly, not all observed lesions were strictly located at the fimbria, stressing the importance of the SEE-FIM protocol, which allows a thorough examination of the entire fallopian tube. Furthermore, the observed abnormalities were not located at the mesothelial–Müllerian junctions, as suggested by some pathologists [46], and no significant pathological aspect was ever found on the ovarian fragments. Thus, removing a part of an ovary attached to the fallopian tube is questionable, from both a pathological and hormonal point of view, as the removal of the fallopian tube only may leave some amount of Müllerian tissue on the ovarian surface [47]. 

Although no pathological aspect was found in the delayed oophorectomy specimens, it is too premature to assume that oophorectomy will not be necessary since the idea that all ovarian HGSCs arise from the fallopian tubes is still controversial [48]. 

We acknowledge possible weaknesses of this study regarding the small sample size and the relatively short current follow-up duration already mentioned. There was no centralized pathological review in this trial, but all participating pathologists were experienced practitioners, fully trained in SEE-FIM protocols. A total of 90% of these pathologists worked in cancer centers and 10% in large private institutions, and they all had access to the national central pathological review if needed. However, this was not required.

In the absence of any scientific validation, our surveillance protocol was based on an annual clinical examination and CA125, followed by an adapted workup if abnormal. It was simple and well accepted and followed by all patients, who had access to information and consultants at any time if they were concerned. 

The lack of protection against de novo or recurrent breast cancer after such a procedure may also be criticized. It is not the main goal of ovarian cancer prophylaxis, and, based on historical cohorts, this advantage of RRSO is currently under question [49], especially for patients with triple-negative tumors. 

## 5. Conclusions

In this pilot study, risk-reducing RF/DO appears to be a safe procedure with similar morbidity to RRSO for patients who refused regular RRSO. The application of the SEE-FIM protocol allowed the diagnosis of a low but objective rate of incidental lesions, possibly missed by a regular tubal examination. However, due to the absence of abnormalities at the mesothelial–Müllerian junctions, a simple bilateral fimbriectomy or salpingectomy may advantageously replace RF. 

We are aware that a larger cohort with longer follow-up is required to confirm our preliminary findings; risk-reducing RF/DO or salpingectomy/DO seems to be a promising prophylactic alternative to RRSO, which prolongs natural ovarian function and, accessorily, fertility. 

## Figures and Tables

**Table 1:** Baseline characteristics (N = 121).

Characteristics		
Age (years)		
Median (range)	39	(28–48)
Performance status, N (%)		
OMS 0	121	(100%)
BMI (kg/m²)		
Median (range)	22.7	(17.6–38.6)
Genetic status, N (%)		
BRCA1 mutation	76	(62.8%)
BRCA2 mutation	31	(25.6%)
Negative or unknown but strong familial history of BOC	14	(11.6%)
Initial biological information, median (range)		
CA125 (MD = 13)	15.1	(4–70.4)
FSH (MD = 12)	5.9	(0–64.3)
Estradiol (MD = 16)	305	(1.9–3186)
Inhibin B (MD = 24)	29	(6–210)
AMH (MD = 30)	5.7	(0.7–81.4)
Family history of breast and ovarian cancer, N (%)		
Any type	110	(90.9%)
Breast cancer only	67	(55.4%)
Ovarian cancer only	7	(5.8%)
Breast and ovarian	36	(29.8%)
Personal history of breast cancer, N (%)		
Overall	69 ^(1)^/121	(57.0%)
In BRCA1-mutation carriers	43/76	(56.6%)
In BRCA2-mutation carriers	20/31	(64.5%)
In patients with no BRCA1/BRCA2 mutation or unknown status	6/14	(42.9%)
Prophylactic bilateral mastectomy before RF, N (%)	30	(24.8%)

MD: number of missing data; ^(1)^ among the 69 patients who had a personal history of breast cancer, 35 had triple-negative breast cancer (32 in BRCA1 mutation carriers, 2 in BRCA2 mutation carriers, and 1 patient with no BRCA1/2 mutation).

**Table 2:** Radical fimbriectomy—surgical data and morbidity (N = 121).

Characteristics		
Surgical approach, N (%)		
Laparoscopic approach	120	(99.2%)
Multiport	66	(54.5%)
Single port	54	(44.6%)
Laparotomy ^(1)^	1	(0.8%)
Abdomino-pelvic exploration, N (%)		
Exploration performed	121	(100%)
Suspicious aspect (adnexas, uterus, peritoneum)	0	(0%)
Peritoneal cytology or washing, N (%)		
Exploration performed	67	(55.4%)
Suspicious aspect	0	(0%)
Technique for transection of ovaries, N (%)		
Scissors + bipolar hemostasis	110	(93.2%)
Endoscopic stapler + bipolar hemostasis	2	(1.7%)
Integrated sealing-section devices	6	(5.1%)
Not specified	3	
Protected specimen extraction, N (%)		
Yes	121	(100%)
Aspect of remaining ovaries at the end, N (%)		
Ischemic	0	(0%)
Reported blood loss		
Yes, N (%)	19	(15.7%)
Volume (mL), median (range)	50	(4–250)
Transfusion, N (%)	0	(0%)
Operative room time (skin to skin) (MD = 14)		
Duration (min), median (range)	45	(18–262)
Duration of hospital stay		
Duration (nights), median (range)	1	(0–31)
Discharge on the day of surgery, N (%)	51	(42.1%)
Per-operative and early post-operative morbidity, N (%)		
Timing	24	(19.8%)
Intraoperative	2	(1.7%)
Early post-operative (in the 30 days)	24	(19.8%)
Any type of adverse event	24	(19.8%)
Grade 2	2	(1.7%)
Grade 1	22	(18.2%)
Pain ^(2)^, Grade 1	13	(10.7%)
Hemorrhage ^(3)^	5	(4.1%)
Grade 2	1	(0.8%)
Grade 1	4	(3.3%)
Nausea/vomiting, Grade 1	5	(4.1%)
Fatigue	2	(1.7%)
Grade 2	1	(0.8%)
Grade 1	1	(0.8%)
Other ^(4)^, Grade 1	9	(7.4%)

^(1)^ RF performed by laparotomy for one patient for whom a concurrent uterine myomectomy was indicated; ^(2)^ pain includes: abdominal pain or discomfort (N = 4), musculoskeletal pain (N = 4), pelvic pain (N = 1), premenstrual pain (N = 1), scar pain (N = 1), bladder pain (N = 1), pain in extremities (N = 1), and pain not otherwise specified (N = 3). ^(3)^ Hemorrhages include two cases of grade 1 per-operative bleeding and three cases of post-operative bleeding. ^(4)^ Other adverse events include: hematoma (N = 2), breast discomfort (N = 1), hot flushes (N = 1), gastrointestinal motility disorder (N = 1), constipation (N = 1), post-procedural inflammation (N = 1), pollakiuria (N = 1), syncope (N = 1), dysmenorrhea (N = 1), keloid scar (N = 1), hyperthermia (N = 1), and eczema (N = 1).

**Table 3:** Pathological results of RF specimens, overall and according to BRCA status.

Pathological Finding on the RF Surgical Specimen	BRCA1 N = 76		BRCA2 N = 31		Negative N = 9		Unknown N = 5		Total N = 121	
	**N**	**%**	**N**	**%**	**N**	**%**	**N**	**%**	**N**	**%**
**Fallopian tube specimen**										
≥1 abnormality	15	19.7%	7	22.6%	3	33.3%	0	0.0%	25	20.7%
HGSC (+STIC)	1	1.3%	0	0.0%	0	0.0%	0	0.0%	1	0.8%
STIC	1	1.3%	0	0.0%	0	0.0%	0	0.0%	1	0.8%
STIL	1	1.3%	0	0.0%	0	0.0%	0	0.0%	1	0.8%
≥1 site of p53 signature with no associated HGSC/STIC/STIL lesion	11	14.5%	4	12.9%	3	33.3%	0	0.0%	18	14.9%
≥1 site with Ki67 > 10% with no associated HGSC/STIC/STIL/p53 lesion	1	1.3%	3	9.7%	0	0.0%	0	0.0%	4	3.3%
No abnormality	61	80.3%	24	77.4%	6	66.7%	5	100.0%	96	79.3%
**Ovarian fragment specimen**										
≥1 abnormality	1 ^(1)^	1.3%	0	0.0%	0	0.0%	0	0.0%	1	0.8%
No abnormality	75	98.7%	31	100.0%	9	100.0%	5	100.0%	120	99.2%

HGSC: high-grade serous carcinoma; STIC: serous tubal intra-epithelial carcinoma; STIL: serous tubal intra-epithelial lesion. ^(1)^ The only abnormality observed in the ovarian fragment specimen was the presence of sites with Ki67 > 10% with no associated HGSC/STIC/STIL/p53 lesion.

## Data Availability

The data presented in this study are available on request from the corresponding author.

## Supplementary Materials

The following supporting information can be downloaded at: https://www.mdpi.com/2072-6694/15/4/1141/s1, English version of the study protocol.

## Clinical Trials Registration

Clinical trial identification number: NCT01608074; date of registration: 29 May 2012; date of initial participant enrollment: 10 January 2012; URL of the registration site: https://clinicaltrials.gov/ct2/show/NCT01608074 (accessed on 30 May 2012).
