# Supplementary material for: Prophylactic Radical Fimbriectomy with Delayed Oophorectomy in Women with a High Risk of Developing an Ovarian Carcinoma: Results of a Prospective National Pilot Study
Source: Cancers (Basel). 2023 Feb 10;15(4):1141. doi: 10.3390/cancers15041141 (PMC9954021; doi:10.3390/cancers15041141)
Supplement: Supplementary file 1 [file cancers-15-01141-s001.zip › cancers-2203213-supplementary.pdf]

[logo:] Oscar Lambret Center
Unicancer HAUTS-DE-FRANCE

STUDY PROTOCOL

Protocol No.: **2011-06**

2011 National Clinical Research Hospital Program

Title:

RADICAL FIMBRIECTOMY FOR YOUNG WOMEN AT HEREDITARY RISK OF PELVIC SEROUS CANCER

Study code: **FIMBRIECTOMIE-1106**

ID-RCB No.: 2011-A01213-38

**Sponsor:**	**Oscar LAMBRET Center**<br>3, rue Frédéric Combemale<br>BP 307<br>59020 LILLE CEDEX<br>Tel.: 03 20 29 59 40 - Fax: 03 20 29 59 71
**Coordinator:**	**Doctor Eric LEBLANC**<br>Department of Gynecological Cancerology<br>email: e-leblanc@o-lambret.fr
**Co-coordinator:**	**Dr. Claude LAVIGNASSE**
**Drafting Committee:**	**Doctor Eric LEBLANC**, M.D.<br>**Doctor Philippe VENNIN,** M.D.<br>**Doctor Nicolas PENEL,** M.D., Ph.D.<br>**Doctor Isabelle FARRE,** M.D.<br>**Yvette VENDEL,** Project Manager<br>**Alicia PROBST**, Project Manager

Confidentiality clause

This document contains information that is the property of the Oscar LAMBRET Center (Centre Oscar LAMBRET, COL) and that is entrusted to you confidentially for review by yourself, your team, the members of the relevant EC and the Administrative Authorities.
The information contained in this document must not be shared with third parties without prior written authorization from the COL, except for the elements necessary to obtain informed consent from those who may participate in the research.

Version No. 3.0 dated 23OCT2019 approved by the Northwest I EC on 10JAN2020 and the ANSM (*Agence nationale de sécurité du médicament et des produits de santé* [French National Agency for Medicines and Health Products Safety]) on 03JAN2020
Version No. 2.0 dated 13DEC2012 approved by the Northwest I EC on 24JAN2013
Version No. 1.1 dated 10NOV2011 approved by the Northwest I EC on 28NOV2011 *(comprising the changes requested by the EC)*
Version No. 1 dated 28SEP2011 approved by the ANSM on 24OCT2011

1 APPROVAL AND SIGNATORIES OF THE PROTOCOL

FIMBRIECTOMIE-1106

Protocol title: *Radical Fimbriectomy for Young Women at Hereditary Risk of Pelvic Serous Cancer*

Date Signature

Doctor Eric LEBLANC /_______________/ /________________________/
Coordinator

Professor Nicolas PENEL
Head of the Department of
Clinical Research and Innovation /_______________/ /________________________/

Professor Eric F. LARTIGAU /_______________/ /________________________/
General Manager

2 PRINCIPAL INVESTIGATOR APPROVAL

I acknowledge that I have read all of the protocol ***No. FIMBRIECTOMIE-1106*** (Protocol No.: 2011-06) and agree to conduct this protocol in accordance with Good Clinical Practice, the Public Health Act of 09AUG2004 and the implementing decree of 26APR2006.

I assume the responsibilities incumbent on me as principal investigator, including:

- the collection of informed consent, signed and dated by patients before any screening procedure in the protocol,
- the validation of the completed case report forms for each of the patients enrolled in the study,
- direct access to source documents for verifications carried out by the monitor appointed by the sponsor,
- archiving of essential study documents for a minimum duration of 15 years.

Name and address of the research site	Name of Principal Investigator	Date and signature

3 LIST OF INVESTIGATORS

Will be attached to the protocol.

4 LIST OF PARTIES INVOLVED

Project coordination<br>***Administrative and regulatory management, monitoring***<br>**Alicia PROBST**, Project Manager<br>**Fabrice MULOT**, Monitor<br>**Caroline DECAMPS**, Administrative Assistant	***Oscar LAMBRET Center***<br>DRCI (*Direction de la recherche clinique et de l'innovation* [Department of Clinical Research and Innovation]) - Promotion Unit<br>Tel.: 03 20 29 59 18 - Fax: 03 20 29 58 96<br>Email: a-probst@o-lambret.fr<br>f-mulot@o-lambret.fr<br>c-decamps@o-lambret.fr<br><br>Promotion@o-lambret.fr
Vigilance<br>**Marie VANSEYMORTIER,** Clinical Trial Vigilance Officer<br>**Julie COURTIAL,** Clinical Trial Vigilance Officer	***Oscar LAMBRET Center***<br>DRCI – Promotion Cell<br>Tel.: 03 20 29 59 18 - Fax: 03 20 29 58 96<br>Email: vigilanceEC@o-lambret.fr
Data Management<br>**Brice DUBOIS**, Head of CTD (*Centre de traitement* [Processing Center])<br>**Valentin HARTER**, Data Manager<br>**Lucie LAROCHE**, Data Manager<br>**Anaïs LELAIDIER**, Data Manager<br>**Romain TRAVERS**, Data Manager	***Northwest Cancer Center Data Processing Center***<br>François Baclesse Center<br>3, avenue du Général Harris<br>14076 CAEN Cedex 05<br>Tel.: 02 31 45 52 87<br>Email: b.dubois@baclesse.unicancer.fr<br>v.harter@baclesse.unicancer.fr<br>laroche.l@baclesse.unicancer.fr<br>a.lelaidier@baclesse.unicancer.fr<br>r.travers@baclesse.unicancer.fr
Data Management and Statistical Analysis<br>**Stéphanie BACQUAERT**, Data Entry Technician<br>**Dr. Marie-Cécile LE DELEY**, Biostatistician Physician Head of the Methodology and Biostatistics Unit<br>**Emmanuelle TRESCH**, Biostatistician	***Oscar LAMBRET Center***<br>DRCI - Methodology and Biostatistics Unit<br>Email: s-bacquaert@o-lambret.fr<br>m-ledeley@o-lambret.fr<br>e-tresch@o-lambret.fr

TABLE OF CONTENTS

1 APPROVAL AND SIGNATORIES OF THE PROTOCOL ____ *2*
2 PRINCIPAL INVESTIGATOR APPROVAL ____ *2*
3 LIST OF INVESTIGATORS ____ *3*
4 LIST OF PARTIES INVOLVED ____ *3*
5 SYNOPSIS ____ *6*
6 INTRODUCTION AND STUDY RATIONALE ____ *10*
6-1 Benefit of radical fimbriectomy [Green 2010] ____ 12
6-2 Potential disadvantages of radical fimbriectomy [Greene 2010] ____ 13
7 STUDY OBJECTIVES ____ *14*
7-1 Primary objective ____ 14
7-2 Secondary objectives ____ 14
8 STUDY DESIGN ____ *14*
8-1 Methodology ____ 14
8-2 Inclusion criteria ____ 14
8-3 Non-inclusion criteria ____ 15
8-4 Early withdrawal criteria ____ 15
8-5 Study withdrawal criteria ____ 15
8-6 Assessment Report ____ 15
8-6-1 Inclusion assessment ____ 15
8-6-3 Follow-up report ____ 17
8-6-4 Study withdrawal assessment ____ 18
8-7 Endpoints ____ 18
8-7-1 Pelvic cancer ____ 18
8-7-2 Occult lesions ____ 19
8-7-3 Incidence or recurrence of breast cancers ____ 19
8-7-4 Incidence of secondary oophorectomy and their morbidity ____ 19
8-7-5 Postoperative complications ____ 19
8-7-5-1 Adverse event ____ 19
8-7-5-2 Serious adverse event ____ 20
8-7-5-3 Expected serious adverse event (expected SAE) ____ 20
8-7-5-4 Unexpected serious adverse event (unexpected SAE) ____ 21
8-7-5-5 Intensity criteria ____ 21
8-7-5-6 Action plan ____ 21
8-7-5-7 SAE follow-up ____ 22
8-7-2-8 Sponsor responsibilities ____ 22
8-7-6 Benign histological abnormalities ____ 23
8-7-7 Satisfaction questionnaire ____ 23
8-8 Statistical methodology ____ 23
8-8-1 Determination of the number of patients and study discontinuation criteria ____ 23
8-8-2 Statistical analysis ____ 23
8-8-3 Data Management ____ 24
9 CONDUCT OF THE STUDY ____ *25*
9-1 Process flow ____ 25
9.1.1 Patient enrollment ____ 25
9.1.2 Surgery: fimbriectomy ____ 25
9-2 Histopathology ____ 27
9-3 "Selectionning [sic: Sectioning] and Extensively Examining the FIMbria" protocol (SEE-FIM) ____ 27
9.3.1 Macroscopic management ____ 27
9.3.2 Histological management ____ 28
9-4 Subsequent processing ____ 28

9.4.1 Incidental finding of invasive cancer 28
9.4.2 Secondary bilateral oophorectomy 29
9-5 Concomitant medication 29
10 REGULATORY AND ETHICAL ASPECTS 29
10.1. Study conduct and sponsor responsibilities (COL) 29
10.2. Study conduct and investigator responsibilities 29
10.3. Ethics Committee (EC) 30
10.4. Participant information and consent 30
10.5. Patient Committee 30
10.6. Independent Monitoring Committee 30
10.7. Confidentiality 31
11 OPERATIONAL MANAGEMENT OF THE STUDY 31
11.1 Study organization 31
11.2. Cost and additional cost of the research 31
11.3. Monitoring 31
11.4. Quality assurance 31
11.5. Ownership of data and publication 31
12 BIBLIOGRAPHIC REFERENCES 32
13- APPENDICES 34
Appendix 1 – Study schedule 34
Appendix 2 – Letter to non-hospital gynecologists 35
Appendix 3 – 2009 Clavien-Dindo classification of surgical complications 36
Appendix 4 – NCI-CTCAE Toxicity Scale Version 4.0 37
Appendix 5 – Patient information sheet and consent form 37
Appendix 6 – List of expected serious adverse events 37
Appendix 7 – Serious adverse event notification forms 37

5 SYNOPSIS

**SPONSOR**	**Oscar LAMBRET Center**<br>3, rue Frédéric Combemale - BP 307 - 59020 LILLE CEDEX
**INDICATION**	Female at hereditary risk of ovarian cancer
**TITLE**	**Radical Fimbriectomy for Young Women at Hereditary Risk of Pelvic Serous Cancer**
**COORDINATOR**<br>**CO-COORDINATOR**	**Doctor Eric LEBLANC**<br>**Doctor Claude LAVIGNASSE**
**NUMBER OF SITES**	14
**TYPE OF STUDY**	Interventional, non-drug, phase II, multicenter study
**OBJECTIVES**	**Primary:**<br>• To assess the incidence of pelvic serous cancer after prophylactic radical fimbriectomy in women at hereditary risk of pelvic serous cancer, but not ready for prophylactic adnexectomy<br><br>**Secondary:**<br>• To assess the morbidity of prophylactic radical fimbriectomy<br>• To determine the proportion of occult lesions on the radical fragmentectomy specimen (in situ and invasive cancer in the tube or adjoining ovary fragment)<br>• To assess the incidence of breast cancers or recurrence of breast cancers after fimbriectomy<br>• To determine the incidence of secondary oophorectomies and their morbidity<br>• To determine the proportion of benign histological abnormalities on radical Fimbriectomy specimens<br>• To assess patient satisfaction later on after the Radical Fimbriectomy
**INCLUSION CRITERIA**	• Women over 35 years of age<br>• After completion of the pregnancy<br>• BRCA 1 or 2 mutation, or family breast/ovarian documented by genealogy<br>• Not ready for bilateral adnexectomy (procedure which will always be routinely offered first)<br>• Involvement or not of breast cancer<br>• Affiliated with a social security plan<br>• Informed consent and signed by the patient prior to implementation of any study-specific procedures
**NON-INCLUSION CRITERIA**	• Postmenopausal female defined as follows:<br>o Bilateral oophorectomy<br>o Not hysterectomized and amenorrheic for more than 12 months and/or FSH >20 IU/L, collapsed estradiol and progesterone, excluding chemo-induced amenorrhea<br>o History of hysterectomy and FSH >20 IU/L<br>o Pregnant and breastfeeding female<br>o Woman under temporary guardianship or guardianship

**METHODOLOGY/NUMBER OF PATIENTS**	The cumulative incidence of pelvic tumors is estimated to be around 10% at 10 years in BRCA mutated women in the absence of prophylactic surgery (Chen et al Meta-analysis of BRCA1 and BRCA2 penetrance JCO 2007; 25:1329-33), corresponding to an "acceptable" rate of 3% at 3 years. A dynamic method for interim analyses for rare events will be used to discontinue the study if, within 3 years, the number of pelvic tumors reaches 2 for 12 participants, 3 for 27 participants, 4 for 46 participants, and 5 for 66 participants for a maximum number of patients of **120*** (Kramar A, Bascoul-Mollevi C. Early Stopping Rules in Clinical Trials Based on Sequential Monitoring of Serious Adverse Events. Med Decis making 2009; 29: 343-350). These decision rules correspond to the comparison of the lower limit of the confidence intervals adjusted on the information fraction and on a concave alpha spending function.<br>Three years after the last patient is enrolled, if 8 pelvic tumors are observed among the 120 patients, the treatment strategy will be considered insufficiently promising as the lower limit of the one sided 90% confidence interval will not contain the acceptable value of 3%.<br><br>*In the end, 123 patients were enrolled in this research; indeed, 3 patients were added to replace patients who had agreed to participate in the study but could not be assessed for the preventive benefit of fimbriectomy (example: non-operated patients)
**STUDY DURATION**	Start of the study: JAN2012<br>Duration of recruitment: 33 months (OCT2014)<br>End of study: AUG2034 (projected)
**ASSESSMENT CRITERIA**	**Primary:**<br>o The cumulative incidence of pelvic cancer will be estimated by measuring the time-to-onset of pelvic cancer, defined as the time from the date of the fimbriectomy to the date of the ovarian cancer, high-grade primary peritoneal, or other gynecologic pelvic location, with death without pelvic cancer considered a competitive event. Study withdrawal for oophorectomy is also a competitive event. Patients alive without pelvic cancer or oophorectomy based on most recent information are censored at this time.<br>**Secondary**:<br>o Morbidity: post-operative complications up to 30 days post-procedure according to Clavien-Dindo classification (2009), according to NCI-CTCAE v4.0 beyond this time frame<br>o Histological occult lesions: number of intraepithelial serous carcinomatous tubal lesions, and invasive cancer on the surgical specimen<br>o Incidence or recurrence of breast cancers: The cumulative incidence of breast cancer or breast cancer recurrence is defined as the time from the date of the fimbriectomy to the date of the breast cancer or recurrence, with death without breast cancer considered a competitive event. Patients alive without breast cancer based on most recent information are censored at this time. A secondary analysis will be performed considering study withdrawal for oophorectomy as a competitive event.<br>o Incidence of secondary oophorectomies and their morbidity:<br>o The cumulative incidence of secondary oophorectomy is defined as the time from the date of the fimbriectomy to the date of the oophorectomy, with death without oophorectomy considered a competitive event. Patients alive without oophorectomy based on most recent information are censored at this time. The timing of the oophorectomy will also be described: prior to menopause, within 6 months of menopause onset, more than 6 months after menopause.<br>o complications according to NCI-CTCAE v4.0 scale

	• Number and type of benign histologic abnormalities in the tube or adjoining ovary specimen<br>• Satisfaction questionnaire
**METHODOLOGY/STATISTICAL CONSIDERATIONS**	The study populations will be as follows:<br>- The descriptive analysis of baseline characteristics will be performed on the patient population enrolled in the study.<br>- The descriptive analysis of the procedure, disease, morbidity, and satisfaction questionnaire will be performed on the patient population who underwent radical fimbriectomy.<br>- The analysis of cumulative incidence of pelvic cancer, breast cancer, and secondary oophorectomy will be performed on the patient population who underwent surgery and excluding patients with invasive cancer or STIC on the fimbriectomy specimen.<br>- The analysis of secondary oophorectomy complications will be performed on the patient population who underwent oophorectomy secondary to the fimbriectomy.<br><br>The statistical analysis of baseline characteristics, procedure, and disease will be essentially descriptive in nature and will be presented in summary tables including the number of missing data. Continuous variables will be summarized by median and extreme values, and mean and standard deviation if warranted. Categorical variables will be presented as contingency tables (absolute frequency and percentage of each modality). The inclusion and exclusion criteria will be verified from the data recorded in the case report forms. Any protocol deviations will be detailed, as well as the causes of ineligibility and non-evaluability. The cumulative incidences of pelvic cancer, breast cancer, and secondary oophorectomy will be estimated by the competitive risk method (Kalbfleish [*sic:* Kalbfleisch] and Prentice). In secondary analysis, cumulative incidences of pelvic cancer and breast cancer will be estimated based on age reached.<br>Early postoperative morbidity (assessed by Clavien-Dindo scale within 30 days post-operatively) and late postoperative morbidity (occurring in a period of more than 30 days and assessed by NCI-CTCAE) will be described by type of adverse event, grade and relationship to the fimbriectomy or secondary oophorectomy. The type of adverse event (AE) will be described by SOC (System Organ Class) and PTname (Preferred Term) of the medDRA medical dictionary. The maximum grades observed by type of AE will be described in tabular form: number and frequency. Serious adverse events (SAE) will be described.<br>Each item of the satisfaction questionnaire will be described by its median, extreme values, mean and standard deviation.

**SHORT DESCRIPTION OF THE METHOD**	The surgical procedure consists of a laparoscopic bilateral radical fimbriectomy. The principle is to remove almost all of the tubes, from the uterine horn (at the level of the uterus because carcinomas develop on the distal part) to the fimbriae, by resecting the part of the ovary adjacent to the ovarian fimbriae (which adhere to the ovary). The purpose of this associated ovarian resection, which justifies its radical name, is to remove the two tube-peritoneal and tube-ovarian junctions, presumed to be the sites of the initial tumor events.<br>This leaves at least 2/3 of the initial ovarian volume in place on each side. The ovaries remain vascularized by their respective infundibulopelvic pedicles. The procedure is performed laparoscopically. An issue concerns hemostasis of the ovary left in place. A pilot series of 10 oophorectomy-completed radical fimbriectomies was performed using all laparoscopic means that combine hemostasis and tissue section. It appears that the use of EndoGIA-type linear staplers is the most effective method. The hemostasis of the ovarian cut section is complete with 3 rows of vascular staples. Sectioning with cold scissors followed by bipolar hemostasis of the remaining ovary part is also effective. Other methods (LigaSure® or Ultracision®) always result, to a greater or lesser extent, in epithelial burning and significantly alter the histological study. In addition, the quality of ovarian hemostasis is often imperfect and its revision leads to thermal damage to the remaining follicles that should be preserved. The routine use of EndoGIA 50 Forceps, vascular staples, is recommended.

6 INTRODUCTION AND STUDY RATIONALE

The identification, by linkage analysis in 1990 and then successive cloning of BRCA1 and BRCA2 genes in 1994 and 1995 disrupted the management of hereditary forms of breast and ovarian cancers. The molecular analysis of both genes now allows identification of a population of women at major risk of these cancers. More than 20,000 consultations and more than 9,000 tests for deleterious BRCA1/2 mutations were performed in France in 2009. Since 2003, the oncogenetic scheme has identified 8,488 people with a BRCA mutation [2009 INCa (*Institut national du Cancer* [National Institute of Cancer]) oncogenetic activity summary].

However, the cancer process remains multifactorial and penetrances are therefore highly variable. The cumulative lifetime risks are around 60 to 90% for breast cancers. For ovarian cancers, these risks are around 40% for BRCA1 and 20% for BRCA2. The mean age of onset is also later for BRCA2. The probability of these cancers occurring before the age of 50 is 5%-10% for BRCA1 and 1%-5% for BRCA2 [Chen 2007; Robson 2007]. The risks vary depending on the number of cases and age of occurrence in families [Metcalfe 2010]. The baseline test is usually performed in an affected person to optimize these tests, which remain complex and expensive with waiting times for results of several months. The predictive test in asymptomatic patients leads to the identification of major breast and ovarian cancer risks and without known equivalent. For breast cancer risk, the majority of women choose specific screening (by MRI) given the physical and psychological consequences of bilateral mastectomy. The situation is different for pelvic risk, as we do not have an effective screening method but a less unacceptable preventive surgical solution than preventive breast surgery, especially in postmenopausal women. The seriousness of ovarian cancers, well known by clinicians as well as women who have had a case in the family, means that most mutated women will opt for preventive adnexal surgery, with or without hysterectomy. It is increasingly clear that adnexal preventive surgery provides the best risk-benefit in this situation with a reduction in both pelvic and breast cancer risk [Domchek 2010].

However, the consequences of castration should be considered in young women. Known over both the short and long-term, they can be improved with estrogenic or estrogen-progestin hormone replacement if the uterus is preserved. Short-term replacement therapy does not seem to prevent reduction significantly in a castration-related breast cancer risk reduction study [Rebbeck 2005]. However, this treatment is contraindicated in women who have previously had breast cancer.

Many mutated women are reluctant to undergo, or delay, ovarian surgery. In the French GENEPSO (*Gène étude prospective sein ovaire* [Ovary breast prospective gene study]) cohort, only 55% of women without disease and 62% of women with breast cancer over 40 years of age opted for adnexal surgery.

The decision for pelvic preventive surgery is therefore particularly difficult in young mutated women, especially for deleterious BRCA1 mutations given the greater pelvic risk, and even more in women who have had breast cancer previously, for whom hormone replacement treatments are contraindicated.

We tried to improve the effectiveness of screening for pelvic tumors by bringing the examinations closer together and performing an ultrasound every 6 months by a trained physician and a CA-125 assay. This study, conducted as part of a PHRC (*programme hospitalier de recherche clinique* [clinical research hospital program]) in 2003, confirmed, consistent with all of the literature on the subject, the lack of utility of screening in these hereditary forms since the two tumors identified out of the 72 assessable patients were peritoneal carcinosis [Taieb 2011].

However, new elements have come to modify the natural history design of ovarian tumors.

Alongside the increase in the number of preventive adnexal surgeries and the increased attention of pathologists to the specimens entrusted to them, it appeared that a significant number of the occult precancerous lesions occurred not on the ovary but on the tube and especially on the uterine horn.

Detailed pathological studies of prophylactic adnexectomy specimens of BRCA1/2 mutated women have revealed the common presence, apart from any invasive process, of serous tubal intraepithelial carcinomatous lesions (or STIC for *Serous Tubal Intraepithelial Carcinoma*) [Lee 2006; Crum 2010]. These abnormalities were also found, adjacent to invasive lesions, in the tubes of patients operated on for sporadic high-grade serous ovarian or peritoneal carcinoma. Another important anatomical feature is that these intraepithelial lesions are mainly found in the fimbrial end of the tube (or **fimbriae**). Immunohistochemical studies of the fimbrial ends of the fallopian tubes in apparently healthy BRCA mutated patients have shown that their cells often have high concentrations of the TP53 protein. This high concentration is caused by a non-functional protein and usually reflects abnormalities in the p53 gene (mutation, overexpression, etc.). This concentration abnormality has been named "signature p53" [Lee 2006]. Its discovery could precede the onset of transitional intraepithelial tubal lesions and STICs [Crum 2010]. However, their specificity is more doubtful because p53 signatures have been found in healthy tubes or even outside the tubes and also in women without a genetic context.

The hypothesis of dissemination of these abnormal tubal cells on the ovarian cortex or in its inclusion cysts, then their development into carcinoma, is supported by very recent biomolecular studies. They indeed confirm the expression by high-grade serous cancer cells of Müllerian markers (such as the tube) and non-mesothelial markers (such as the ovarian cortex) [Kurman 2010]. This would therefore be a matter of serous high-grade carcinomas, of secondary carcinogenesis of the ovary through the tube, and not primary ovarian tumor. According to Kurman, type 1 lesions would thus originate in the carcinogenesis of post-ovulation inclusion cysts, which, when healing the ovarian defect, would therefore include Müllerian tissue. The type of carcinoma then depends on the origin of these Müllerian cells. Thus, inclusion cysts with tubal-derived cells may cause low-grade serous carcinoma, either from the outset or after a stage of serous borderline tumor. If the cells are of endometrial origin (directly by menstrual blood reflux, or indirectly through endometrial injury), they may produce endometrioid carcinomas or some clear cell carcinomas [Kobayashi

2009]. Cells of endocervical origin may also cause clear cell carcinomas. Finally, although a more hypothetical mechanism, paratubal cells, located at the junction between the fimbriae and ovarian epithelium, would give rise to mucinous carcinomas and degenerated Brenner tumors, which would otherwise be precursors [Kurman 2010].

The various forms of ovarian carcinoma could therefore be categorized into two main anatomic/clinical and prognostic types [Shih 2004]. Schematically, type 1 would be slow-growing tumors, so more often found in the early stages. It includes low-grade serous, mucinous, grade 1 to 2 endometrioid and clear cell carcinomas. These tumors have specific molecular abnormalities, all of which focus on mitotic signaling or cell survival pathways (KRAS (mucinous), BRAF, HER2 (low serous grade), PTEN and beta-catenin (endometrioid), PI3K-AKT (clear cells) [Gilks 2010; Despierre 2010]. They have stable genetics and few p53 mutations in common. In contrast, type 2 tumors are high-grade serous or endometrioid carcinomas [Geyer 2009], carcinosarcomas, and undifferentiated carcinomas. These lesions usually progress rapidly, despite a good initial response to medical treatment. At the biomolecular level, they only very rarely have abnormalities in mitotic signal transduction or survival, but present from the outset with various genetic alterations of the p53 gene, causing an ineffective protein to trigger the repair of altered DNA or, failing that, cellular apoptosis. These differences thus suggest different origins and development modalities of these 2 tumor types. It is hypothesized that type 2 tumors mostly originate in the tube and are now rather grouped together as **serous pelvic carcinoma** that no longer indicates their ovarian or primary peritoneal tubal origin. **Most of the pelvic tumors associated with BRCA1/2 would be type 2. This reminds us that pathologists have long been perplexed about the origin of ovarian tumors, which have so far had no identified precursor and studies that have shown, at least for some, a reduction in pelvic risk through tubal ligation. These facts would also explain the ineffectiveness of screening for these initially tubal and rapidly ovarian and peritoneal proliferative tumors.**

6-1 Benefit of radical fimbriectomy [Green 2010]

- The discovery of a macroscopic cancer abnormality during prophylactic adnexal surgery is a rare possibility, but the frequency of invasive or in situ occult lesions identified on operative specimens ranges from 2 to 10%. The rate of cancerous and precancerous lesions observed on the fallopian tubes of preventive surgery specimens for hereditary risk varies from 50 to 100%. These proportions vary depending on the study population (identified mutation, suspected, “high risk” of ovarian cancer, history of breast cancer) by age at the time of the procedure and of course according to the microscopic analysis protocol, which is now well codified. If the exact proportion remains inaccurate, it is accepted that some serous pelvic carcinomas related to BRCA1/2 mutations are of tubal origin and may potentially be prevented by this surgery. If the intramural part of the tube never has abnormalities or degeneration, the interstitial portion and especially the region of the fimbrial end (fimbriae) is the most common place for the

development of these tubal lesions. The most recent developments focus on epithelial junctions (specifically Müllerian mesothelium-epithelium), firstly the junction between the Müllerian mucous membrane of the fimbrial end and the tubal peritoneal serosa (mesothelium), and secondly, the junction between the mucous membrane of the fimbrial end and the ovarian surface mesothelium [Auersperg 2010; Rabban 2010, Seidman 2010].

- The proposed surgical procedure removes, bilaterally, the entire tube starting at the level of the uterus and taking the portion of the ovary adhering to fimbriae. This "radicality" toward the ovary thus ensures the complete excision of these fimbriae and their junction with the ovary, which will also be analyzed in detail.
- If surgery enables bilateral removal of the portion of the ovary adhering to the tube, the remaining ovary is sufficient to maintain enough hormonal activity and avoid complications of early and sudden menopause.
- In almost all cases, this surgery will be performed laparoscopically (see Surgical Technique, chapter 9.1.2) with limited risks of surgical complications.
- This procedure, limited to the tubes, may be more acceptable for young women who are often unwilling to cope with the consequences of early and permanent menopause.
- This solution also allows for a potential, even theoretical, preservation of fertility since medically assisted reproduction techniques could be considered later in these cases.

6-2 Potential disadvantages of radical fimbriectomy [Greene 2010]

- The rate of pelvic tumors that develop from the tube is not precisely known, and the benefit for these women will obviously be proportional.
- The idea is to perform castration afterward, closer to the natural age of menopause, and this will require a second pelvic procedure. Some women may not want to consider this second surgery, in the hope of a sufficiently reduced risk by removing the tube.
- The potential drawback that seems most important to us is the lack of reduction in breast cancer risk through ovarian suppression. Castration is generally known to reduce the risk of breast cancer, particularly tumors with steroid receptors. It is more than likely that this risk reduction is through ovarian hormone suppression. The studies confirm this risk reduction for women who have a BRCA1 and BRCA2 mutation [Rebbeck 2009]. However, it should be noted that in prospective studies this reduction in breast cancer risk is greater for BRCA2 mutations than BRCA1, which is not unexpected given the large proportion of tumors without BRCA1 receptors. However, the absence of a receptor may not necessarily reflect complete independence of the tumor from sex hormones. It should also be noted that these studies are observational and subject to risks of bias that are not always easy to identify or correct; therefore, the estimate of breast cancer risk reduction is only approximate [Heermskerk 2010]. One can also argue about the possibility of replacement therapy after castration; however, this treatment is contraindicated in breast cancer and some studies show that this treatment may not be as

effective as natural hormonal impregnation [Rebbeck 2009]. A substantial proportion of women who will be enrolled will likely have a history of BRCA1-related HR- breast cancer and the influence of castration on contralateral cancer risk is not really documented [Domchek 2010].

All of these arguments justify a prospective study of bilateral radical fimbriectomy that may help to better understand the risk-benefit ratio in this complex and very personal choice, but also to better understand the natural history of high-grade pelvic tumors that are obviously not just the prerogative of BRCA 1/2 mutations.

7 STUDY OBJECTIVES

7-1 Primary objective

- To assess the incidence of pelvic serous cancer after prophylactic radical fimbriectomy in women at hereditary risk of pelvic serous cancer, but not ready for prophylactic adnexectomy

7-2 Secondary objectives

- To assess the morbidity of prophylactic radical fimbriectomy
- To determine the proportion of occult lesions on the radical fragmentectomy specimen (in situ and invasive cancer in the tube or adjoining ovary fragment)
- To assess the incidence of breast cancers or recurrence of breast cancers after fimbriectomy
- To determine the incidence of secondary oophorectomies and their morbidity
- To determine the incidence of benign histological abnormalities on radical Fimbriectomy specimens
- To assess patient satisfaction at a distance from radical Fimbriectomy

8 STUDY DESIGN

8-1 Methodology

This is a phase II, multicenter, non-drug, interventional biomedical study that assesses the feasibility of radical fimbriectomy in young women at hereditary risk of ovarian cancer.

8-2 Inclusion criteria

These are women in whom preventive adnexectomy is usually recommended.

All criteria are required for inclusion:

- Women over 35 years of age
- After completion of the pregnancy
- BRCA 1 or 2 mutation, or family breast/ovarian documented by genealogy
- Not ready for bilateral adnexectomy (procedure that will always be routinely offered 1st)
- Involvement or not of breast cancer
- Affiliated with a social security plan

- Informed consent and signed by the patient prior to implementation of any study-specific procedures

8-3 Non-inclusion criteria

Only one criterion is sufficient for non-inclusion:

- Postmenopausal female defined as follows:
  - Bilateral oophorectomy
  - Not hysterectomized and amenorrheic for more than 12 months and/or FSH >20 IU/L, collapsed estradiol and progesterone, excluding chemo-induced amenorrhea
  - History of hysterectomy and FSH >20 IU/L
- Pregnant or breastfeeding woman
- Woman under temporary guardianship or guardianship

8-4 Early withdrawal criteria

- Discovery of invasive cancer or serous intraepithelial carcinomatous tubal lesion (or STIC for *Serous Tubal Intraepithelial Carcinoma*) on the fimbriectomy specimen

8-5 Study withdrawal criteria

Patients will be followed until menopause (no periods for more than 1 year).

The criteria for withdrawal from the study are:

- Post-fimbriectomy oophorectomy (patients will be followed 1 month post-procedure for complications)
- Patient decision
- Patient lost to follow-up
- Patient death

8-6 Assessment Report

8-6-1 Inclusion assessment

Written consent from the patient must be obtained prior to implementation of any study-specific procedures. Patients eligible for the trial must undergo a baseline assessment within 28 days before surgery. At the same time, the relevant history (risk factors, associated diseases) will be recorded.

Type of Assessment	Timeline - Comments
Information and collection or written consent	**To be obtained and signed by the patient and the investigator**
Demographics<br>Relevant history (risk factors, associated diseases)<br>Last menstrual period date	Prior to enrollment<br>and within 28 days prior to surgery
**PHYSICAL EXAMINATION**<br>Gynecological examination<br>Anesthetic evaluation of surgical non-contraindication	
**LABORATORY TESTS**<br>- FSH, estradiol, inhibin B and anti-Müllerian hormone (ovarian reserve)<br>- CA-125	
- β-HCG	Before enrollment and within **7 days** prior to surgery

8-6-2 Post-operative assessment

Type of Assessment	Timeline - Comments
**PHYSICAL EXAMINATION**<br>- Physical examination<br>- Postoperative Complications	30 days after surgery

8-6-3 Follow-up report

The primary objective of radical fimbriectomy is to reduce the risk of serous pelvic carcinoma (ovarian or peritoneal) with a predominant tubal origin, while awaiting definitive castration.

Since a large portion of the ovaries is left in place, there is a residual risk of high-grade serous carcinoma that is probably very low but not zero. It could be due to the degeneration of STIC tissue left in place on the ovary (but which would also exist on the fimbriectomy specimen) or by the degeneration of inclusion cysts [Kurman, 2010]. Finally, the development of any other type 1 tumor can be observed from tissue included in these same cysts or a non-epithelial tumor, germ cell tumor, or sex cords that is not related to the BRCA gene abnormality.

Any abnormal abdominal or gynecological symptoms, whether there is a genetic background or not, should result in appropriate imaging being performed, possibly repeated or supplemented by surgery. The follow-up of these asymptomatic women at risk should also be considered.

Surveillance methods have proved ineffective at screening for high-grade serous carcinoma. However, they make more sense when it comes to detecting a type 1 tumor or a non-epithelial tumor that usually grows more slowly.

To date, the screening combining the annual CA-125 blood test followed-up according to its transvaginal ultrasound result, appears to be the combination that leads to the least "unnecessary" surgeries while detecting most early cancers, as shown in the largest randomized screening study conducted to date [Menon 2009].

For other women without lesions identified on the fimbriectomy specimens, we thus propose to base the pelvic monitoring procedures of these women on this protocol. This gynecological screening will be combined with breast disease screening.

If a test is abnormal, it is repeated. In case of persistent abnormality, further clinical assessment (MRI) or surgical follow-up will be discussed.

Type of Assessment	Timeline - Comments
**PHYSICAL EXAMINATION**<br>Gynecological examination<br>Additional examination left to clinician discretion	Once a year
**LABORATORY TESTS**<br>- FSH, estradiol, inhibin B and anti-Müllerian hormone (ovarian reserve)	In case of menstrual disorders<br>or menopausal symptoms
- Annual CA-125	Once a year
**PARACLINICAL EXAMINATIONS**<br>- Transvaginal and suprapubic ultrasound<br>- ± pelvic MRI if clinical signs	Pelvic ultrasound, if marker increase confirmed by a second sample
**QUESTIONNAIRE**<br>- Satisfaction questionnaire	Once during follow-up

The investigator must inform the non-hospital gynecologist of the patient's participation in the trial, and the need to investigate, at each consultation:

- any adverse event that may reasonably be related to surgery, such as menstrual disorders, hot flashes, vaginal dryness, etc., until the onset of menopause;
- any signs suggestive of pelvic cancer, breast cancer, or its recurrence;
- and any additional pelvic surgery procedure

A letter for non-hospital gynecologists is provided for this purpose (Appendix 2).

8-6-4 Study withdrawal assessment

Type of Assessment	Timeline - Comments
**PHYSICAL EXAMINATION**<br>Gynecological examination<br>Additional examination left to clinician discretion	
**LABORATORY TESTS**<br>- FSH, estradiol, inhibin B and anti-Müllerian hormone (ovarian reserve)	In case of menstrual disorders<br>or menopausal symptoms
- CA-125	Single assay

8-7 Endpoints

8-7-1 Pelvic cancer

The cumulative incidence of pelvic cancer will be estimated by measuring the time-to-onset of pelvic cancer, defined as time from the date of the fimbriectomy to the date of the ovarian cancer, high-grade primary peritoneal cancer, or cancer of other gynecologic pelvic location, with death without pelvic cancer considered a competitive event. Study withdrawal for oophorectomy is also a competitive event. Patients alive without pelvic cancer or oophorectomy based on most recent information are censored at this time.

8-7-2 Occult lesions

The proportion of occult lesions on the fimbriectomy specimen is defined as the number of intraepithelial serous carcinomatous tubal lesions, and in situ or invasive cancer on the surgical specimen (at the fallopian tube or adjoining ovary fragment).

The proportion of p53 signature foci and proliferative foci (ki67 ≥ 10%) will also be described.

8-7-3 Incidence or recurrence of breast cancers

The cumulative incidence of breast cancer or breast cancer recurrence is defined as the time from the date of the fimbriectomy to the date of the breast cancer or recurrence, with death without breast cancer considered a competitive event. Patients alive without pelvic cancer or oophorectomy based on most recent information are censored at this time. A secondary analysis will be performed considering study withdrawal for oophorectomy as a competitive event.

8-7-4 Incidence of secondary oophorectomy and their morbidity

Their morbidity is also theoretical and very low. It involves possible gastrointestinal or ureteral injury related to the release of adhesions between the remaining ovaries and these structures as well as bleeding from the adnexal pedicle. The greatest risk is actually the development of ovarian cysts on the remaining ovaries, as the theoretical age of menopause approaches. However, this risk is not greater than that of the general population. It is the significance of these cysts in this particular context that should contribute to whether or not specific surgery is indicated. Complications related to secondary oophorectomy will be assessed up to one month post-procedure according to NCI-CTCAE v4.0.

The cumulative incidence of secondary oophorectomy is defined as the time from the date of the fimbriectomy to the date of the oophorectomy, with death without oophorectomy considered a competitive event. Patients alive without pelvic cancer or oophorectomy based on most recent information are censored at this time. The timing of the oophorectomy will also be described: prior to menopause, within 6 months of menopause onset, more than 6 months after menopause.

8-7-5 Postoperative complications

Morbidity will be assessed:

- up to 30 days post-procedure according to the Clavien-Dindo classification [2009]
- and beyond this time (until confirmation of menopause for patients who have not had a secondary oophorectomy, or up to one month after secondary oophorectomy) according to the NCI-CTCAE v4.0 grading scale.

8-7-5-1 Adverse event

An adverse event is any change in the patient’s initial state before treatment, including intercurrent diseases, whether or not the event is considered to be related to the products studied and occurring at any time during the study.

The nature of each adverse event, date of onset, duration, seriousness, relationship to surgery, associated treatments, and outcome will be established. The intensity of adverse events will be graded according to the criteria based on their time to onset (see above).

8-7-5-2 Serious adverse event

Not considered a serious adverse event (SAE):

- Hospitalization scheduled prior to the start of the trial and/or planned per protocol (biopsy etc.).

- Definition:

A serious adverse event (SAE) is any event:

- Leading to death,
- Life-threatening,
- Leading to hospitalization or prolongation of hospitalization,
- Causing permanent disability or severe temporary disability,
- Causing a birth defect, fetal impairment or abortion,
- Medically significant.

The terms *disability and incapacity* are any temporary or permanent physical or psychological disability that is clinically significant and affects the patient's physical activity and/or quality of life.

Any *medically significant* clinical event or laboratory finding considered serious by the investigator that does not meet the severity criteria defined above. They may pose a risk to the patient and require medical intervention to prevent an outcome corresponding to one of the severity criteria mentioned above *(examples: overdoses, second cancers, pregnancies and new facts that may be considered medically significant*).

Patient death by progression is an expected serious event.

8-7-5-3 Expected serious adverse event (expected SAE)

An expected SAE is an event already mentioned in the literature data.

Perioperative morbidity from prophylactic laparoscopic adnexectomy is very low. The rates of intraoperative complications were reported at 1.3% and postoperative complications at 3.1% with a **conversion** rate **to laparotomy** of 0.8% in a series of 159 patients [Kenkhuis 2010]. There is no theoretical reason why fimbriectomy has higher levels than this. Apart from the known risks of any laparoscopic procedure, the specific operative morbidity of radical fimbriectomy would be limited to the sole risk of **postoperative bleeding** of the remaining ovaries. An inspection of the ovarian cut section after deflation of the pneumoperitoneum over a few minutes and re-insufflation enables sealing with a few elective bipolar coagulations if necessary.

Later, the **risk of induced menopause** may theoretically be observed if the two sources of blood supply from the ovary (adnexal and infundibulopelvic pedicle) were altered by the procedure. This risk is naturally increased in case of a single adnexa. If menopause is confirmed by the absence of periods

and a hormone panel (LH and FSH plasma estradiol), the additional oophorectomy could be discussed with the patient sooner.

8-7-5-4 Unexpected serious adverse event (unexpected SAE)

An unexpected SAE is an event that is not mentioned or different in nature, intensity, or progression from what is described above.

8-7-5-5 Intensity criteria

The intensity criterion should not be confused with the severity criteria, which serve as a guide for defining reporting obligations.

The intensity of events will be estimated according to the Clavien-Dindo and NCI-CTC version 4.0 (grade 1 to 5 toxicity) classifications.

The intensity of adverse events not listed in this classification will be assessed according to the following qualifications:

Mild (Grade 1):	does not affect the patient's usual daily activity
Moderate (Grade 2):	interferes with the patient's usual daily activity
Severe (Grade 3):	prevents the patient's usual daily activity
Very Severe (grade 4):	imposes resuscitation measures/life-threatening
Death (Grade 5)	

8-7-5-6 Action plan

The investigator informs the Sponsor of all Expected Serious Adverse Events (expected SAE) and Unexpected Serious Adverse Events (unexpected SAE), whether or not they are attributable to the research, that occur during the study or within 30 days after the fimbriectomy.

All delayed Serious Adverse Events (occurring after this 30-day period) considered as being reasonably related to the research must be reported without time limitation.

The notification is made by email using the *"Serious Adverse Event Notification"* form recorded as accurately as possible, signed and dated.

Email: vigilanceEC@o-lambret.fr

In case of malfunction or any other issue preventing emailing (and only in this case), the form can be faxed to the sponsor at 03 20 29 58 96.

The investigator will note in particular for each event:

- Its description as clearly as possible according to the medical terminology,
- The intensity,
- The start and end date of the event,
- The measures taken and whether or not corrective treatment is necessary,
- Its clinical course. In case of a non-fatal event, progression should be followed until recovery

or return to the previous state or stabilization of any sequelae,

- Its cause, and in particular the causal relationship between this event and the study or a research-related constraint (additional examinations requested as part of the research, etc.).

The investigator should also attach to the serious adverse event report, whenever possible:

- A copy of the hospitalization or prolongation of hospitalization report,
- A copy of all the results of additional tests performed, including relevant negative results with normal laboratory values,
- Any other document he/she deems useful and relevant.

All these documents must be anonymized.
Additional information may be requested (by fax, phone, email, or visit) by the sponsor.

8-7-5-7 SAE follow-up

The investigator is responsible for appropriate medical follow-up of the patients until resolution or stabilization of the event or death of the patient. This may sometimes mean that this follow-up will continue after the patient has withdrawn from the trial.

He/she forwards the additional information to the Sponsor using an SAE notification form (by checking the Follow-up No. X box to clarify that this is a follow-up and not an initial report). He/she also forwards the last follow-up upon resolution or stabilization of the SAE.
He/she keeps the documents regarding the presumed SAE in order to allow, if necessary, the previously sent information to be completed.

He/she responds to requests for additional information from the Sponsor to document the initial observation.

8-7-2-8 Sponsor responsibilities

➢ Recording of pharmacovigilance data and immediate reporting of unexpected SAEs

The sponsor will be responsible for maintaining and storing all study-related vigilance data.
The sponsor will also be responsible for reporting all suspected unexpected serious adverse reactions (SUSAR) to the ANSM, the relevant EC and for informing the investigators in accordance with applicable regulations.

➢ New facts

As with all research involving human subjects, the sponsor will send to the investigators concerned any information likely to affect the safety of subjects (Art. R1123-52 CSP (*Code de la santé publique* [French Public Health Code])) and will immediately inform the competent authority and the ethics committee of new facts1 and, if applicable, the measures taken (Art. R1123-59 of the CSP).

➢ Periodic safety reports

[1] **New fact:** any new data that could lead to a reassessment of the risk-benefit ratio or the research product of the research, changes in the use of this product, in the conduct of the research, or documents relating to the research, or could suspend or interrupt or modify the research protocol or similar research studies (CSP, Art. R1123-46, 12°).

The sponsor is responsible for preparing and drafting periodic safety reports and submitting them to the ANSM and relevant EC in accordance with applicable regulations.

8-7-6 Benign histological abnormalities

- Number and type of benign histologic abnormalities in the tube or adjoining ovary specimen

8-7-7 Satisfaction questionnaire

A questionnaire will be given to the patients who have had surgery, to give them a voice about their feelings and their satisfaction. This questionnaire consists of 5 items to assess:

- patient concerns about their decision to opt for a fimbriectomy – and, if applicable, oophorectomy before menopause age;
- the respective symptoms associated with these interventions,
- and if patients would be willing to recommend fimbriectomy if this technique was clinically validated.

8-8 Statistical methodology

8-8-1 Determination of the number of patients and study discontinuation criteria

The cumulative incidence of pelvic tumors is estimated to be around 10% at 10 years in BRCA mutated women in the absence of prophylactic surgery (Chen et al Meta-analysis of BRCA1 and BRCA2 penetrance JCO 2007; 25:1329-33), corresponding to an "acceptable" rate of 3% at 3 years. A dynamic method for interim analyses for rare events will be used to discontinue the study if, within 3 years, the number of pelvic tumors reaches 2 for 12 participants, 3 for 27 participants, 4 for 46 participants, and 5 for 66 participants for a maximum number of patients of 120 (Kramar A, Bascoul-Mollevi C. Early Stopping Rules in Clinical Trials Based on Sequential Monitoring of Serious Events. Med Decis making 2009;29:343-350). These decision rules correspond to the comparison of the lower limit of the confidence intervals adjusted on the information fraction and on a concave alpha spending function.

Three years after the last patient is enrolled, if 8 pelvic tumors are observed among the 120 patients, the treatment strategy will be considered insufficiently promising as the lower limit of the one sided 90% confidence interval will not contain the acceptable value of 3%.

8-8-2 Statistical analysis

The statistical analyses will be performed by the Oscar Lambret Center Methodology and Biostatistics Unit (*Unité de Méthodologie et de Biostatistique*, UMB).

The study populations will be as follows:

- The descriptive analysis of baseline characteristics will be performed on the patient population enrolled in the study.
- The descriptive analysis of the procedure, disease, morbidity, and satisfaction questionnaire will be performed on the patient population who underwent radical fimbriectomy.

- The analysis of cumulative incidence of pelvic cancer, breast cancer, and secondary oophorectomy will be performed on the patient population who underwent surgery and excluding patients with invasive cancer or STIC on the fimbriectomy specimen.
- The analysis of secondary oophorectomy complications will be performed on the patient population who underwent oophorectomy secondary to the fimbriectomy.

The statistical analysis of baseline characteristics, procedure, and disease will be essentially descriptive in nature and will be presented in summary tables including the number of missing data. Continuous variables will be summarized by median and extreme values, and mean and standard deviation if warranted. Categorical variables will be presented as contingency tables (absolute frequency and percentage of each modality).
The inclusion and exclusion criteria will be verified from the data recorded in the case report forms. Any protocol deviations will be detailed, as well as the causes of ineligibility and non-evaluability.

The cumulative incidences of pelvic cancer, breast cancer, and secondary oophorectomy will be estimated by the competitive risk method (Kalbfleish [*sic:* Kalbfleisch] and Prentice), as defined in section 8.7 (endpoints).
In secondary analysis, cumulative incidences of pelvic cancer and breast cancer will be estimated based on age reached.
Early postoperative morbidity (assessed by Clavien-Dindo scale within 30 days post-operatively) and late postoperative morbidity (occurring in a period of more than 30 days and assessed by NCI-CTCAE) will be described by type of adverse event, grade and relationship to the fimbriectomy or secondary oophorectomy. The type of adverse event (AE) will be described by SOC (System Organ Class) and PTname (Prefered Term) of the medDRA medical dictionary. The maximum grades observed by type of AE will be described in tabular form: number and frequency. Serious adverse events (SAE) will be described.

Each item of the satisfaction questionnaire will be described by its median, extreme values, mean and standard deviation.

8-8-3 Data Management

Data management will be performed in collaboration with the Northwest Cancer Center Data Processing Center (CTD (*Centre de traitement des données* [Data Processing Center])-CNO (*Cancéropôle nord-ouest* [Northwest Cancer Center]), approved by the INCa. A study-specific database will be created using the Capture System (CLINSIGHT) and tested and validated prior to entry.
A data validation plan will be developed and will describe in detail the controls to be executed for each variable as well as the list of allowable obvious corrections. The data will be monitored by the data management team using error messages from validation programs. The obvious errors will be corrected. The other errors, omissions or inconsistencies will be mentioned on the correction request forms, which

will be sent to the study investigator for resolution. The corrections will be included by the Clinical Research Associate Monitor (CRA-M) in the database.

Special case

The data related to benign histological abnormalities and satisfaction questionnaire will be collected in an Excel spreadsheet, and hosted securely by the Methodology and Biostatistics Unit, before being included in the final analysis.

The database will be frozen after final quality control and then exported to STATA statistical software using an automated and validated procedure.

9 CONDUCT OF THE STUDY

9-1 Process flow

9.1.1 Patient enrollment

An enrollment request form will be completed by the investigator to ensure that the patient meets ALL screening criteria. The investigator must give the information letter to the patient and have the document signed (consent) allowing the subject to express his/her willingness. PRIOR TO SURGERY, the investigator must fax the enrollment request form to the Study Coordination Center:

Integrated Clinical Research Unit/Promotion Unit
of the Oscar Lambret Center – Lille
Fax: 03.20.29.58.96

After verification of all screening criteria, an identification number will be assigned. A confirmation of enrollment will be sent to the study doctor.

9.1.2 Surgery: fimbriectomy

The surgical procedure consists of a laparoscopic bilateral radical fimbriectomy. The principle is to resect almost all of the tubes, from the uterine horn (at the level of the uterus because carcinomas develop on the distal part) to the fimbriae, by resecting the part of the ovary adjacent to the ovarian fimbriae (which adhere to the ovary). This associated ovarian resection, which justifies the term "radical", has 2 objectives:

- The first, real, is to ensure resection of the entire fallopian tube fimbrial end and tubal-ovarian adhesion.
- The second is to resect the fimbria/ovarian junction, the area that appears most sensitive to the development of serous lesions [Auersberg 2010; Seidman 2010] and, more hypothetical, to remove this transitional epithelium from which Brenner tumors and some mucinous carcinomas would develop [Kurman 2010].

This leaves at least 4/5 of the initial ovarian volume in place on each side. The ovaries remain vascularized by their respective utero-ovarian pedicles. The procedure is performed laparoscopically.

In accordance with national guidance regarding adnexal prophylactic surgery (August 2009 on www.e-cancer.fr), peritoneal cytology and systematic peritoneal biopsies (excluding suspicious lesion) will not be performed.

<u>The laparoscopy</u> is set up as for a classic bilateral adnexectomy:

- Ventilated intubated patient with empty bladder and stomach, supine, arms along the body.
- 15 to 20° Trendelenburg position applied after insertion of the optic.

Pneumoperitoneum performed according to the patients by puncture in the upper left quadrant of the abdomen or by open laparoscopy. Blind umbilical punctures should be avoided in favor of left subcostal puncture.

- Four trocars are put in place:
  - 2 x 10 mm trocars, 1 umbilical for the optic and 1 medial suprapubic for instruments and protected specimen extraction
  - and 2 x 5 mm trocars placed in the iliac fossae outside the epigastric pedicles for the instruments. This can also be done using the single [site] umbilical trocar technology (cosmetic advantage).
  - **<u>Variant</u>**: The use of a single umbilical trocar (Gelpoint® Applied Med type) is possible. With this device, specimens can be extracted directly through the umbilicus, and through the gel, since the wall is protected.

After careful exploration of the abdominopelvic cavity, and in the absence of a suspicious abnormality, <u>the fimbriectomy</u> itself is performed:

- It begins by separating the tube from the uterus, flush with it.
- The bipolar coagulation of the tube will prevent any bleeding.
- The underlying utero-ovarian pedicle should not be coagulated because it will become the only vascular collateral of the part of the ovary left in place.
- The mesosalpinx sectioning is then performed with scissors as close as possible to the interstitial portion of the tube so as not to injure the vascular arch over the ovary.
- At the fimbrial end of fimbriae, the ovary is gently grasped and its section proceeds beyond the insertion of the utero-ovarian ligament.
- During this procedure, all or part of the pelvic infundibular pedicle must be sectioned after hemostasis.

The procedure is of course bilateral.

In case of a single adnexa (e.g., history of salpingectomy for ectopic pregnancy), the procedure will be unilateral without the need to resect the contralateral isolated ovary (unless abnormal).

There is apparently no difference in technical difficulty on one side or the other, except for the need, on the left, as with conventional adnexectomy, to pre-release possible adhesions between the sigmoid colon and the adnexa.
A biopsy will be performed if a pelvic macroscopic abnormality is discovered.
The extraction of the fimbriectomy specimens will be carried out in separate endoscopic bags (specifying which side is sampled).

We conducted a comparative study of the different means of sectioning and ovarian hemostasis [Leblanc 2011]:

- the use of cold scissors with additional non-routine bipolar hemostasis or the use of EndoGIA® 45mm Forceps (Autosuture Tyco Healthcare France) vascular staples are the most effective methods for hemostasis and tissue alterations.
- The bipolar-scissor combination has our preference over EndoGIA® that is less comfortable to use (especially if single trocar) and is of a significant cost.
- Note: A video explaining the procedure will be sent to the participating sites. One of the surgeons at the Coordinating Center will also be available for any on-site assistance if necessary.

9-2 Histopathology

The detection of early occult lesions within prophylactic bilateral adnexectomies performed in BRCA mutated women requires detailed and rigorous sampling of these adnexas. This meticulous approach shows that these small lesions, often less than 1 mm in size, are more commonly located in the tubal infundibulum rather than in the ovary.
The pathological study of operative specimens should optimize the possibility of detecting early carcinoma.
The tubes and ovarian portions covered by the tube should be studied in their entirety with a more careful study of the fibrial end. The ideal dissection protocol should optimize visualization of the mucous membrane of the fibrial end of the tube and surface epithelium of the ovary.

9-3 "Selectionning [*sic:* Sectioning] and Extensively Examining the FIMbria" protocol (SEE-FIM)

The surgical specimens are sent fresh to the pathology laboratory of each participating site.

9.3.1 Macroscopic management

Tubes: after separating the fimbrial end and the infundibulum from the ampulla and isthmus❶, the fimbrial end and the infundibulum are cut longitudinally ❷ (in the direction of the length of the fimbrial end fimbriae). The sections are preferably placed in 2 cassettes to spread the tube fimbriae properly. The rest of the tube is cut into sections perpendicular to its axis❸.

Ovarian portions covered by the tube: each ovary fragment is sectioned perpendicular to its longitudinal axis❹.

To enable detection of lesions of 1 millimeter, the cut sections should not exceed, if possible, a thickness of 2 to 3 mm. Under these conditions, each adnexa represents approximately 10 to 15 cassettes.

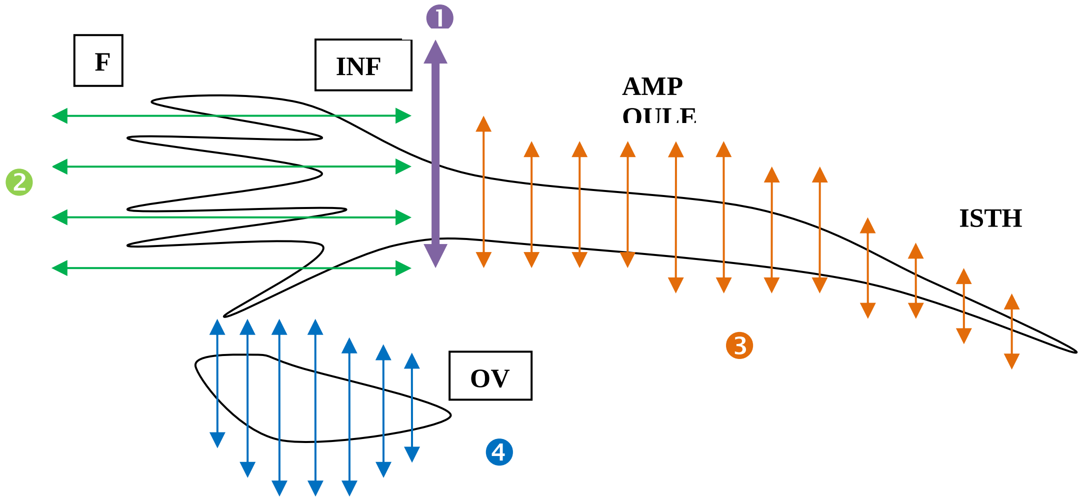

9.3.2 Histological management

- For each block of tube, 1 ribbon of 7 representative sections is made.
- Standard Hematein-Phloxin-Safran (HPS) staining is performed routinely (and first-line) as well as first-line immunohistochemistry with anti-p53 and anti-ki67 antibodies.
- The other 4 sections are kept in reserve.
- The performance of first-line serial sections is unnecessary: these will be requested later by the pathologist in case of diagnostic doubt.

- For each block of ovary, 1 ribbon of 7 sections is made.
- These ribbons are stored after HPS staining on one of the sections of each ribbon.

The immunohistochemical study is not routine and may later be requested by the pathologist based on histological findings on the HPS.

9-4 Subsequent processing

9.4.1 Incidental finding of invasive cancer

In case of incidental discovery of invasive ovarian or peritoneal tubal carcinoma, a further procedure will be scheduled that will follow the surgical and medical management rules for invasive ovarian carcinoma developed by the National Cancer Institute guidelines team in 2009 (documents available at: www.e-cancer.fr).

Otherwise, pelvic monitoring is started, the procedures of which are described below.

9.4.2 Secondary bilateral oophorectomy

Secondary bilateral oophorectomy will be performed solely after release of potential periovarian adhesions by coagulation and sectioning of the pedicle and utero-ovarian ligament. The broad ligament of the uterus approach (through opening of the peritoneum along the external iliac pedicle) may be necessary in case of adhesions to this pedicle or if the infundibulopelvic ligament has been preserved. In these cases, checking the position of the ureter will avoid subsequent complications. They will also be extracted through endoscopic bags.

9-5 Concomitant medication

Not applicable.

10 REGULATORY AND ETHICAL ASPECTS

10.1. Study conduct and sponsor responsibilities (COL)

The study must be conducted in accordance with the ethical principles of the Declaration of Helsinki, Good Clinical Practice (GCP) of the International Conference of Harmonization (ICH-E6), Law 2004-800 of 06AUG2004 on bioethics and the provisions of the Public Health Act of 09AUG2004 and the implementing decree of 26APR2006.

The sponsor must ensure essential documents on the conduct of the study are stored under conditions ensuring their safety, for the minimum duration provided by GCP, i.e., 15 years after the end of the research.

10.2. Study conduct and investigator responsibilities

The principal investigator at each institution undertakes to conduct the clinical trial in accordance with the protocol that has been approved by the EC and ANSM. The investigator cannot deliberately carry out a protocol deviation without the authorization of the EC and the ANSM.

It is the responsibility of the principal investigator:

- to provide the sponsor with his/her *curriculum vitae* as well as those of the co-investigators,
- to identify the members of his/her team participating in the trial and to define their responsibilities,
- to start recruiting patients after authorization from the sponsor,
- to make every effort to enroll the required number of patients within the established recruitment period.

It is the responsibility of each investigator:

- to collect the informed consent personally signed and dated by the patient before any trial-specific screening procedure,

- to regularly complete the case report forms (eCRFs) for each patient enrolled in the trial and to allow the Clinical Research Associate Monitor (CRA-M) direct access to the source documents so that the latter can validate the CRF data,
- to date, correct and validate the eCRF corrections for each patient enrolled in the study,
- to accept the regular visits of the CRA monitor, and possibly those of auditors mandated by the sponsor or inspectors of the supervisory authorities.

All documentation relating to the study (protocol, consents, CRF, investigator file, etc.), as well as the original documents (laboratory results, radiology, consultation reports, clinical examination reports prepared, etc.) must be kept in a secure location and considered confidential material.

The archiving of data will be under the responsibility of the investigator and according to current legislation. The investigator will keep the data and a patient identification list for a minimum of 15 years after the end of the study.

10.3. Ethics Committee (EC)

In accordance with the applicable regulations, the clinical study protocol and the various amendments are submitted by the study sponsor for the opinion of an EC.

10.4. Participant information and consent

Prior to the conduct of biomedical research on a subject, the **free, informed consent** of the subject must be collected after he/she has been informed of the purpose of the research, the conduct and duration of the study, the potential benefits, risks and constraints of the study, and the opinion given by the EC and ANSM.

The consent form will be personally signed and dated by the patient and the investigator (original filed by the investigator, a copy will be given to the patient).

10.5. Patient Committee

The protocol will be reviewed by the Patient Committee of the National League Against Cancer (*Ligue Nationale Contre le Cancer*, LNCC) particularly focusing on the quality of the information letter, provision of a treatment and monitoring plan and the suggestion of measures to improve patient comfort.

10.6. Independent Monitoring Committee

The establishment of an independent trial monitoring committee will be set up to ensure the protection of patients, to ensure that the trial is conducted ethically, to assess the risk-benefit ratio of the trial and to ensure the review of the scientific findings during and at the end of the trial. The Monitoring

Committee will meet mid-study and then, if applicable, in case of high incidence of pelvic cancers, during the monitoring period of patients.

It will consist of 3 members:

- 1 biostatistician: **Sylvie CHABAUD**, Léon Bérard Center, Lyon
- 1 surgeon: **Prof. Philippe MORICE**, Gustave Roussy Institute, Villejuif
- 1 oncogenesis specialist: **Dr. Sophie Lejeune**, Jeanne de Flandre Hospital, Lille CHRU (*Centre hospitalier régional universitaire* [Regional University Hospital])

10.7. Confidentiality

In accordance with the French Public Health Code, the investigators and all persons called to collaborate in the study are required to maintain confidentiality with respect to the study, the participants and the findings obtained. The investigator must ensure that the anonymity of patients is respected. The investigator maintains a confidential patient identification list.

11 OPERATIONAL MANAGEMENT OF THE STUDY

11.1 Study organization

This study is sponsored by the Hauts-de-France Cancer Center: the Oscar LAMBRET Center of Lille (COL).

11.2. Cost and additional cost of the research

Any additional costs related to activities that are not routinely performed are the subject of an agreement negotiated between the COL and the investigator site representative, taking into account the financial resources available to the COL as part of its public promotion activity.

However, the COL ensures the organization and logistics (protocol, electronic case report form, investigator file) necessary for the conduct of the study.

11.3. Monitoring

Initiation visits or telephone meetings, follow-up visits, and close-out visits or letters are carried out by the CRA monitor appointed by the sponsor, in accordance with GCP.

11.4. Quality assurance

The Sponsor is responsible for the implementation and maintenance of a quality assurance system to ensure that the study is conducted in accordance with the protocol and GCP.

11.5. Ownership of data and publication

At the end of the study, a report will be written by the sponsor and then validated by the coordinator.

No publication or presentation of the results of this trial may be made without the sponsor's consent.

Any publication shall at a minimum refer to:

- the coordinating investigator,

- all those who have participated in the study. The order of co-authors will take into account the participation of the various investigators in the trial (number of patients enrolled and assessable).
- all those who have contributed significantly to the conduct of the study and the publication process: the CRA monitor, the project manager, the biostatistician, the head of the Clinical Research and Innovation Department, and the sponsor.

Furthermore, any publication will include thanks to:

- Participating site research technicians,
- INCa (PHRC 2011),
- Study related partners (CTD-CNO)

The sponsor's review and agreement are required before any communication.

12 BIBLIOGRAPHIC REFERENCES

1. Auersperg N. The origin of ovarian carcinoma: a unifying hyppothesis. Int J Gynecol Pathol 2010; 30: 12-21.
2. Chen S, Parmigiani G. Meta-analysis of BRCA1 and BRCA2 penetrance. J Clin Oncol 2007; 25: 1329-33.
3. Crum CP, Drapkin R, Miron A, Ince TA, Muto M, Kindelberger DW, Lee Y. The distal fallopian tube: a new model for pelvic serous carcinogenesis. Curr Opin Obstet Gynecol 2007; 19: 3-9.
4. Clavien PA, Barkun J, de Oliveira ML et al. The Clavien-Dindo classification of surgical complications: five-year experience. Ann Surg. 2009 Aug;250(2):187-96
5. Despierre E, Lambrechts D, Neven P et al. The molecular genetic basis of ovarian cancer and its roadmap towards a better treatment. Gynecol Oncol 2010; 117: 358-365.
6. Domchek SM, Friebel TM, Singer CF et al. Association of risk-reducing surgery in BRCA1 or BRCA2 mutation carriers with cancer risk and mortality. JAMA 2010; 304: 967-75.
7. Geyer JT, Lopez-Garcia MA, Sanchez-Estevez C et al. Pathogenetic pathways in ovarian endometrioid adenocarcinoma: a molecular study of 29 cases. Am J Surg Pathol 2009; 33: 1157-63.
8. Gilks C. Molecular Abnormalities in Ovarian Cancer Subtypes Other than High-Grade Serous Carcinoma. J Oncol 2010.
9. Greene MH, Mai PL, Schwartz PE. Does bilateral salpingectomy with ovarian retention warrant consideration as a temporary bridge to risk-reducing bilateral oophorectomy in BRCA1/2 mutation carriers? Am J Obstet Gynecol 2010.
10. Kenkhuis M, de Bock G, Oude Elferink P et al. Short-term surgical outcome and safety of risk reducing salpingo-oophorectomy in BRCA1/2 mutation carriers. Maturitas 2010.
11. Kobayashi H, Kajiwara H, Kanayama S et al. Molecular pathogenesis of endometriosis-associated clear cell carcinoma of the ovary (review). Oncol Rep 2009; 22: 233-40.
12. Kramar A, Bascoul-Mollevi C. Early stopping rules in clinical trials based on sequential monitoring of serious adverse events. Med Decis Making 2009; 29: 343-50.
13. Kurman RJ, Shih Ie M. The origin and pathogenesis of epithelial ovarian cancer: a proposed unifying theory. Am J Surg Pathol 2010; 34: 433-43.
14. Lee Y, Medeiros F, Kindelberger D et al. Advances in the recognition of tubal intraepithelial carcinoma: applications to cancer screening and the pathogenesis of ovarian cancer. Adv Anat Pathol 2006; 13: 1-7.
15. Madalinska JB, van Beurden M, Bleiker EM et al. The impact of hormone replacement therapy on menopausal symptoms in younger high-risk women after prophylactic salpingo-oophorectomy. J Clin Oncol 2006; 24: 3576-82.
16. Menon U, Gentry-Maharaj A, Hallett R et al. Sensitivity and specificity of multimodal and ultrasound screening for ovarian cancer, and stage distribution of detected cancers: results of the prevalence screen of the UK Collaborative Trial of Ovarian Cancer Screening (UKCTOCS). Lancet Oncol 2009; 10: 327-40.

17. Metcalfe K, Lubinski J, Lynch HT et al. Family History of Cancer and Cancer Risks in Women with BRCA1 or BRCA2 Mutations. J Natl Cancer Inst 2010; 102: 1874-78.
18. Rebbeck TR, Friebel T, Wagner T et al. Effect of short-term hormone replacement therapy on breast cancer risk reduction after bilateral prophylactic oophorectomy in BRCA1 and BRCA2 mutation carriers: the PROSE Study Group. J Clin Oncol 2005; 23: 7804-10.
19. Rebbeck TR, Kauff ND, Domchek SM. Meta-analysis of risk reduction estimates associated with risk-reducing salpingo-oophorectomy in BRCA1 or BRCA2 mutation carriers. J Natl Cancer Inst 2009; 101: 80-7.
20. Robson M. Is breast conservation a reasonable option for women with BRCA-associated breast cancer? Nat Clin Pract Oncol 2007; 4: 10-1.
21. Seidman JD, Yemelyanova A, Zaino RJ, Kurman RJ. The Fallopian Tube-Peritoneal Junction: A Potential Site of Carcinogenesis. Int J Gynecol Pathol 2010.
22. Shih Ie M, Kurman RJ. Ovarian tumorigenesis: a proposed model based on morphological and molecular genetic analysis. Am J Pathol 2004; 164: 1511-8.
23. Taïeb S., Rocourt N, Narducci F et al. Inefficacy of screening pelvic tumours for hereditary risk of ovarian neoplasms. Bull Cancer, 2011; 98(2): 113-9
24. Leblanc E, Narducci F, Farre I, Peyrat JP et al: Radical fimbriectomy: areasonable temporary ris-reducing surgery for selected women with germ line mutation of BRCA 1 or 2 genes? Rationale and preliminary development. Gynecol Oncol 2011; 121: 472-6

13- APPENDICES

Appendix 1 – Study schedule

EXAMINATIONS	Prior to enrollment and within 28 days prior to fimbriectomy	30 days after fimbriectomy	Once a year	Withdrawal from the study (3)
Consent signature	X			
Inclusion criteria	X			
Exclusion criteria	X			
**PHYSICAL EXAMINATION**<br>Gynecological examination	X	X	X	X
Anesthetic evaluation of surgical non-contraindication	X			
Postoperative Complications		X		
**LABORATORY TESTS**<br>• FSH, Estradiol	X		X (1)	X (1)
• LH				X
• Inhibin B<br>• Anti-Müllerian Hormone (ovarian reserve)	X		X (1)	X (1)
• CA-125	X		X	X
• βHCG	X (within 7 days before surgery)			
**PARACLINICAL EXAMINATIONS**<br>• Endovaginal and supravaginal ultrasound<br>• +/- Pelvic MRI			X (2)	X (2)

(1) If you have menstrual problems or symptoms of menopause
(2) Transvaginal and supravaginal ultrasound in case of increased marker confirmed by a second sample +/- pelvic MRI in case of clinical signs
(3) See chapter 8.6.4

Appendix 2 – Letter to non-hospital gynecologists

Doctor ....

On ...

Subject: ***FIMBRIECTOMIE-1106 clinical study***

Patient's last name and first name: ....

Date of birth: ⎿__⊥__⏌ ⎿__⊥__⏌ ⎿__⊥__⊥__⊥__⏌
Patient's initials: ⎿__⏌– ⎿__⏌ **Patient Number:** ⎿__⊥__⊥__⏌

Dear Colleague,

We are currently participating in a clinical study sponsored by the Oscar Lambret Center entitled ***Radical fimbriectomy for young women at hereditary risk of serous pelvic cancer*** (FIMBRIECTOMIE-1106). This is a study in which the above-named patient agreed to participate and underwent a fimbriectomy on ....

The rationale for this study is based on the increased risk of women with deleterious BRCA1/2 mutations developing breast or ovarian cancer, often reluctant to undergo adnexal surgery. The pelvic risk is even greater in women who have previously had breast cancer. Based on data from the literature, pathology findings reveal that most occult precancerous lesions occur not on the ovary but on the tube and especially on the fimbrial end of the fallopian tube. Its objective is to assess the incidence of pelvic serous cancer after prophylactic radical fimbriectomy in women at hereditary risk of pelvic serous cancer, but not ready for prophylactic adnexectomy. This trial also aims to determine the occult lesion rate on the fimbriectomy specimen, the incidence or recurrence of breast cancers, the rate of secondary oophorectomy and [its] morbidity, and postoperative complications.

In this study, patients should receive annual monitoring until menopause (breast-gynecological examination). Any menopausal symptoms or menstrual disturbances should trigger routine laboratory tests as per the study protocol (FSH, estradiol, inhibin B, AMH, and CA-125).

This is why we would be grateful if you could let us know:

- of any adverse event that could occur in this patient: signs and symptoms that may be reasonably related to surgery, menstrual disorders, hot flashes, vaginal dryness, etc.,
- the occurrence of pelvic cancer, breast cancer,
- and any additional pelvic surgery procedure.

If you have any questions or would like more information, please contact the investigator site in charge of monitoring this patient (see contact details below).

Thank you for your help. Sincerely,

Name, date and signature

Appendix 3 – 2009 Clavien-Dindo classification of surgical complications

This classification is used for surgical complications that occur up to 30 days after surgery.

**Grades**	**Definition**
**I**	Any deviation/divergence from normal postoperative follow-up not requiring pharmacological or surgical treatment, or endoscopic or radiological intervention
	Allowable treatments are: drugs such as antiemetics, antipyretics, analgesics, diuretics, as well as electrolytes and physiotherapy.<br>This grade also includes infected injuries.
**II**	Requiring pharmacological treatment with drugs other than those authorized for grade I complications<br>Transfusions and parenteral nutrition are also included in this grade.
**III**	Requiring a surgical procedure, endoscopic or radiological
III-a	Without general anesthesia
III-b	With general anesthesia
**IV**	Life-threatening complication (including central nervous system complications) whether or not requiring hospitalization in intensive care
IV-a	Single organ dysfunction (including dialysis)
IV-b	Multiple organ dysfunction
**V**	Patient death
**"d" suffix**	If the patient has a complication at the time of discharge, "d" suffix (for "disability") is added to the respective grade of the complication. This label indicates the need to track the event in order to assess the complication fully.

Appendix 4 – NCI-CTCAE Toxicity Scale Version 4.0

http://safetyprofiler-ctep.nci.nih.gov/CTC/CTC.aspx

Appendix 5 – Patient information sheet and consent form

Will be attached to the protocol

Appendix 6 – List of expected serious adverse events

Type of complications	Operative complications	Postoperative complications
Gastrointestinal	• Intestinal or colonic wound	• Nausea, vomiting<br>• Constipation<br>• Obstruction<br>• Fistula
Thromboembolic		• Deep Venous Thrombosis<br>• Pulmonary embolism
Hemorrhagic	• Arterial, venous wound<br>• Bleeding<br>• Transfusion	• Bleeding<br>• Transfusion<br>• Wall hematoma
Lymphovascular		• Lower extremity edema<br>• Lymphedema (1)<br>• Lymphocele
Urine	• Bladder wound<br>• Ureteral wound	• Fistula<br>• Stenosis<br>• Bladder disorders (2)
Infectious		• Urinary tract infection<br>• Abscess<br>• Secondary infection (abscess, lymphocele)<br>• Sepsis at surgical site or not
Neurological	• Nerve wound (obturator, femoral, genitofemoral nerve involvement)<br>• Ureteral wound	• Obturating femoral cutaneous neuralgia<br>• Paresthesia, lower extremity dysesthesia<br>• Lower extremity mobility disorder (adduction disorder)
Other	• Anaphylactic shock<br>• Quincke's edema	• Anaphylactic shock<br>• Umbilical hernia<br>• Eventration, complicated or not<br>• Pain (abdominopelvic)<br>• Fatigue, heaviness, weight<br>• Menopause

Appendix 7 – Serious adverse event notification forms

Will be attached to the protocol
